# Connectome disruptions after hypoxic-ischaemic injury associate with consciousness disorder severity

**DOI:** 10.1093/braincomms/fcag117

**Published:** 2026-04-02

**Authors:** Sofia G Hilger, Eric S Rosenthal, Annelise M Kulpanowski, Jacob A Dodelson, Gaston Cudemus-Deseda, Marjorie Villien, Brian L Edlow, James L Januzzi, MingMing Ning, W Taylor Kimberly, Edilberto Amorim, M Brandon Westover, William A Copen, Pamela W Schaefer, Joseph T Giacino, David M Greer, Ona Wu

**Affiliations:** Athinoula A. Martinos Center for Biomedical Imaging, Department of Radiology, Massachusetts General Hospital, Harvard Medical School, Charlestown, MA 02129, USA; Department of Neurology, Massachusetts General Hospital, Boston, MA 02114, USA; Athinoula A. Martinos Center for Biomedical Imaging, Department of Radiology, Massachusetts General Hospital, Harvard Medical School, Charlestown, MA 02129, USA; Athinoula A. Martinos Center for Biomedical Imaging, Department of Radiology, Massachusetts General Hospital, Harvard Medical School, Charlestown, MA 02129, USA; Department of Cardiac Anesthesiology and Critical Care Medicine, Massachusetts General Hospital, Boston, MA 02114, USA; Athinoula A. Martinos Center for Biomedical Imaging, Department of Radiology, Massachusetts General Hospital, Harvard Medical School, Charlestown, MA 02129, USA; Athinoula A. Martinos Center for Biomedical Imaging, Department of Radiology, Massachusetts General Hospital, Harvard Medical School, Charlestown, MA 02129, USA; Department of Neurology, Massachusetts General Hospital, Boston, MA 02114, USA; Department of Medicine, Cardiology Division, Massachusetts General Hospital and Baim Institute for Clinical Research, Boston, MA 02114, USA; Department of Neurology, Massachusetts General Hospital, Boston, MA 02114, USA; Department of Neurology, Massachusetts General Hospital, Boston, MA 02114, USA; Department of Neurology, Massachusetts General Hospital, Boston, MA 02114, USA; Beth Israel Deaconess Medical Center, Boston, MA 02215, USA; Department of Radiology, Neuroradiology Division, Massachusetts General Hospital, Boston, MA 02114, USA; Department of Radiology, Neuroradiology Division, Massachusetts General Hospital, Boston, MA 02114, USA; Department of Physical Medicine and Rehabilitation, Spaulding Rehabilitation Hospital, Harvard Medical School, Charlestown, MA 02129, USA; Department of Neurology, Boston University School of Medicine, Boston Medical Center, Boston, MA 02118, USA; Athinoula A. Martinos Center for Biomedical Imaging, Department of Radiology, Massachusetts General Hospital, Harvard Medical School, Charlestown, MA 02129, USA

**Keywords:** hypoxic ischaemic brain injury, structural connectome, disorders of consciousness, diffusion MRI, brain connectivity

## Abstract

Accurate neuroprognostication of cardiac arrest survivors who are initially comatose after restoration of spontaneous circulation is crucial for guiding patient management. Because hypoxic-ischaemic injury is typically diffuse, damage to a network of brain regions is likely involved in the patient’s disorder of consciousness. To quantify these complex brain network changes, graph theoretical methods were applied. We hypothesize that structural connectivity metrics may provide insights into which patients will likely recover consciousness. Eighteen comatose patients (50 ± 22 years, 44% male) and four healthy participants (40 ± 20 years, 50% male) underwent multi-shell high angular diffusion MRI as part of a prospective study. Structural connectivity matrices were constructed using probabilistic tractography to measure the likelihood of connections between anatomical regions. Network topology alterations were quantified using clustering coefficient, global efficiency and degree. Hub index analysis was performed to explore the impact of anoxic injury on high-degree hubs. Network parameters were compared between patients with arousal recovery (AR, eye-opening to auditory or noxious stimulation) and without arousal recovery (No AR). Analyses were repeated for AR patients who achieved emergence from the minimally conscious state (EMCS) within one-year post-cardiac arrest and AR patients who did not achieve EMCS (AR′). Significant differences were observed between the Controls, AR and No AR for all four metrics (Kruskal–Wallis Tests, *P* < 0.05). Worsening disorders of consciousness were associated with decreasing brain complexity (Kendall's tau, *P*<0.01). Post-hoc testing showed Control values were significantly greater than No AR for all metrics (Wilcoxon rank sum, *P* < 0.05). Control values were greater than AR for all metrics (*P* < 0.05), except the clustering coefficient (*P* = 0.36). AR was significantly greater than No AR for all metrics (*P* < 0.05), except for the hub index (*P* = 0.12). Notable differences between AR′ and Controls were observed for all metrics (*P* < 0.05), except clustering coefficient (*P* = 0.11). No significant differences were found between AR′ and No AR groups. In contrast, for all metrics, EMCS values were not significantly different compared with the Controls but were significantly different than the No AR cohort values (*P* < 0.05). The hub index analysis revealed disproportionate damage to high-degree nodes such as the thalamus, putamen and precuneus, further linking topological disruption to the severity of outcomes. This study highlights the potential of graph theoretical measures of structural connectivity to guide decisions in the care of comatose cardiac arrest patients. By bridging structural connectivity with clinical outcomes, this research provides valuable insights into the neural mechanisms underlying consciousness and recovery after cardiac arrest.

## Teaser

Hilger *et al*. investigated how structural brain connections are altered after hypoxic-ischaemic brain injury. Patients who regained consciousness preserved complex structural network topology. In contrast, patients exhibiting network disruptions in efficiency and hub regions had worse outcomes. Network-based biomarkers may aid in prognosis and understanding of consciousness recovery.

## Graphical Abstract

**Graphical Abstract:**
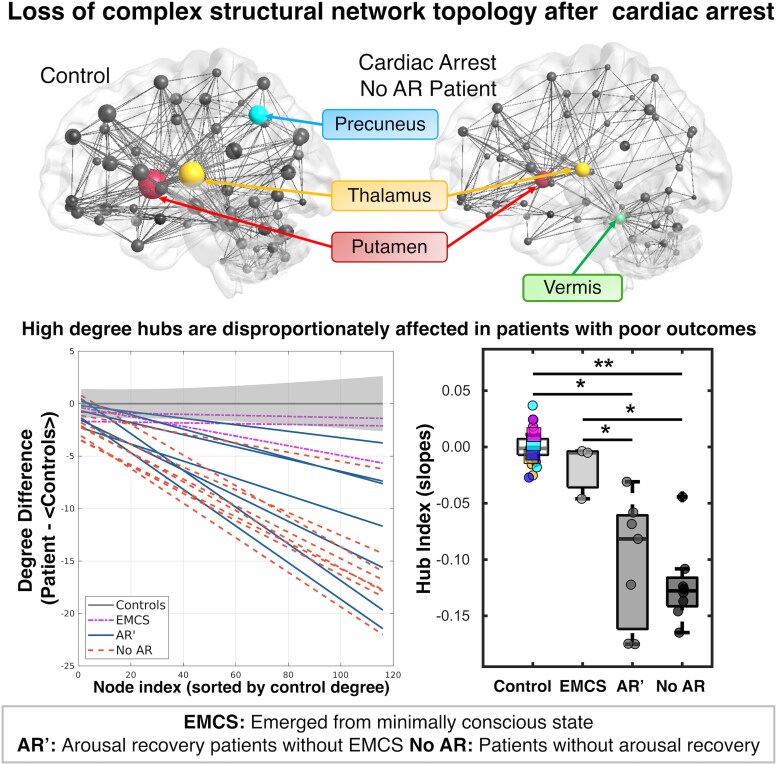

## Introduction

Accurate neuroprognostication of outcomes for patients who are comatose due to cardiac arrest (CA) is essential. Patients who survive CA experience varying degrees of cognitive impairment or a range of disorders of consciousness (DOC) due to diffuse hypoxic ischaemic brain injury (HIBI).^1,2^ Advances in post-resuscitation care have reduced initial mortality but thereby also leave a greater proportion of patients with uncertain neurological prognoses.^3^ Early prognostication is difficult except in extreme cases: patients who awaken rapidly do well, and those with extensive HIBI do poorly, but intermediate conditions require cautious interpretation of multimodal data and often extended observation, with an unclear long-term prognosis.^3^ During the initial 72 h following CA, sedation often obscures clinical evaluations by delaying awakening neurological responses, making it challenging to distinguish between patients with transient coma who may recover and those with persistent coma who are unlikely to improve.^4-6^ Accurate prognoses are vital for guiding critical decisions about continuing or withdrawing life-sustaining treatment (WLST) and helping clinicians and families balance the potential for recovery against the risks of prolonged suffering or futile care.^7^

Recovery of consciousness following CA may occur over an extended period,^8^ with cognitive recovery often progressing independent of motor recovery. This dissociation can cause underestimation of a patient's underlying cognitive abilities.^9^ Advances in neuroimaging have enabled detection of signs of awareness and rudimentary communication in patients diagnosed clinically with unresponsive wakefulness syndrome.^10^ Recent reports of delayed recoveries further complicate prognostication, as some patients achieve favourable neurological outcomes beyond standard recommended observation windows.^11,12^

At present, no single predictor or combination of predictors can ensure accurate prognostication.^1,7,13^ Current recommendations advocate for a cautious, multimodal approach combining neurologic exams, blood biomarkers, neuroimaging and electrophysiological tools.^13^ While some early indicators predict poor outcomes within a week, variability and bias persist.^14^ Furthermore, the behavioural diagnostic criteria that determine the boundaries of syndromes of unconsciousness remain indistinct, and misdiagnosis is common.^15,16^ Studies in this field are further affected by biases, particularly from early WLST.^6,7^

The extent of HIBI after cardiac arrest is a primary determinant of neurologic outcomes in hospitalized survivors.^17^ The severity of the resulting DOC is associated with both the extent of widespread diffuse anoxic injury^18,19^ and focal damage to the putamen^20,21^ and thalamus.^22^ In patients with DOC, cerebral dysfunction has been associated with disconnection,^23^ with functional recovery tied to preserved underlying structural integrity.^24^ Quantitative metrics based on graph network theory may thus be particularly useful for measuring structural disconnection disorders.^25^ Graph theoretical methods have been used to study complex networks, including the brain, and have been applied to structural and functional brain connectivity networks to uncover underlying organization.^26^ Brain networks in healthy individuals have been shown to exhibit small-world properties,^27,28^ characterized by high global efficiency (short paths between nodes) and high clustering, indicating tightly knit groups. These two properties balance segregation (local clustering and specialization) and integration (global communication efficiency).^29,30^ We hypothesize that patients who recover consciousness will exhibit greater global efficiency and clustering than patients who do not. To test our hypothesis, we conducted a prospective pilot observational study in the acute care setting of cardiac arrest patients who remained comatose after restoration of spontaneous circulation (ROSC).

## Materials and methods

### Participants

Twenty-two adult cardiac arrest patients were prospectively enrolled between December 2013 and April 2018. Patients were enrolled who met the following criteria: (i) age ≥18 years, (ii) post-ROSC Glasgow Coma Scale^31^ (GCS) score ≤8, (iii) no MRI contraindications and (iv) no pre-arrest neurocognitive disorders. Protocol amendments are available in Supplementary Methods. Informed consent was obtained from all participants or their legally authorized representatives. A healthy control cohort of five adults (age ≥18, no known cognitive disorders or MRI contraindications) was also enrolled (Controls). One patient and one control were excluded due to differences in the number of slices acquired for the diffusion MRI (Supplementary Fig. 1). Patient characteristics have been previously reported in a task-based functional MRI investigation.^32^ With consent, patient-level task-based functional MRI results had been shared with the clinical team, with confounders like motion or sedation discussed during reporting.^32^ However, patient-level results of structural connectivity analyses were not shared with the clinical teams.

### Neurological testing and outcome assessments

Demographic, clinical characteristics (e.g. initial cardiac rhythm, hospital admission GCS) and targeted temperature management details were obtained from medical records. The initial rhythms were categorized as shockable (ventricular tachycardia or ventricular fibrillation) or non-shockable (pulseless electrical activity or asystole). Favourable short-term neurological outcome was defined as arousal recovery (AR), indicated by eye opening to verbal or noxious stimulation by hospital discharge. Modified Rankin Scale (mRS) and Cerebral Performance Category (CPC) scores were recorded at discharge and at 3-month, 6-month and 1-year follow-ups. Missing CPC and mRS values were carried forward from prior visits. Functional outcomes for survivors were assessed using the CRS-R,^33^ with good long-term outcomes defined as achieving emergence from the minimally conscious state (EMCS) (score of 2 on the Communication Scale or 6 on the Motor Function Scale) within 1 year.

### Data acquisition

All participants underwent scans on a 3T MRI system (Skyra, Siemens Medical Solution) located in the neurosciences intensive care unit. High-resolution 3D T1-weighted images (3DT1WI, multi-echo magnetization-prepared gradient-echo) were obtained for registration purposes, with a field of view (FOV) of 256 × 256 mm^2^, an acquisition matrix of 256 × 256, and 176 1 mm thick sagittal slices. Multi-shell diffusion imaging was performed by acquiring blipped simultaneous multi-slice^34^ echo-planar imaging (slice acceleration factor = 2, iPAT = 2) in 30 bipolar gradient directions with b-values of 1000 s/mm^2^ and 2000s/mm^2^ (voxel size: 3 × 3 × 3 mm^3^, 72 × 72 in-plane acquisition matrix and 50 slices, repetition time ranging from 4000 to 4200 ms, echo time 96 ms), along with 10 b = 0 s/mm^2^ images, with a total acquisition time of 5:08 (5 min 8 s) to 5:23.

### Brain network analysis

Graph theory methods are increasingly being used to understand complex biological systems, including the human brain organization in both health and disease.^26^ Complex networks exhibit small-world properties,^27,28^ characterized by high global efficiency (short paths between nodes) noted in random networks and high clustering (indicating tightly knit groups) noted in regular lattice networks. These two properties balance segregation (local clustering) and integration (global communication efficiency).^29,30^ The small-world architecture of brain networks forms a crucial foundation for theories underpinning the conscious experience, particularly the brain’s capacity to integrate specialized processing areas^35^ and to broadcast information in a conscious ‘workspace’.^36^ Specialized processing relies on clustering, allowing efficient local communication, while integration across distinct processing areas depends on high-degree hub nodes.

### Network construction

**Figure 1:**
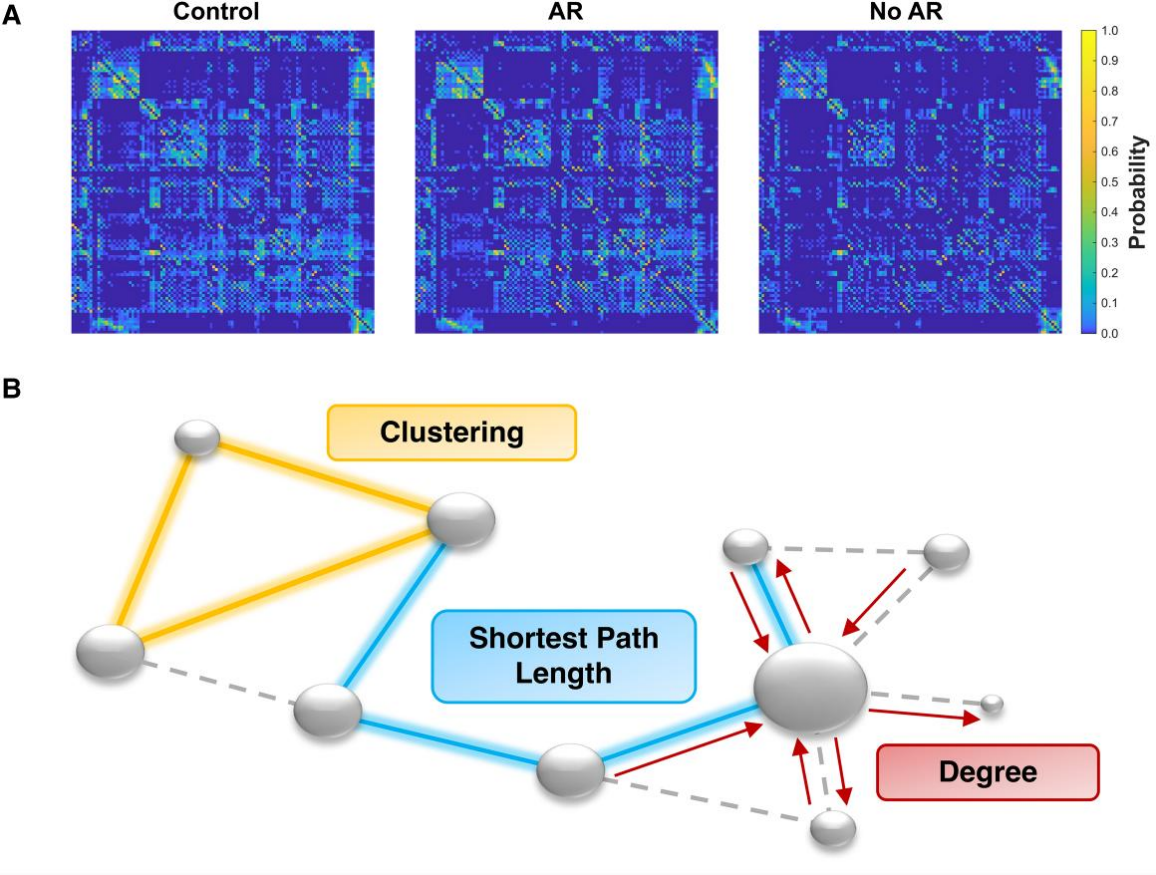
**Structural brain networks. (A)** Raw association matrices are shown for a Control participant, an AR patient and a No AR patient. The probabilistic weight of each connection is expressed as a colour. **(B)** A visual representation of graph theoretical measures: the clustering coefficient for the nodes connected to the yellow triangle is one (all their neighbours are interconnected); the shortest path length highlighted is four; the degree for the highlighted hub has in-degree value of four and out-degree value of three, resulting in a total degree of seven. Degree is reflected by the size of the nodes, and the dashed lines represent other nonzero edges between nodes. Global efficiency is the inverse of the shortest path length. AR: Arousal Recovery; No AR: No arousal recovery.

Network nodes were defined based on the Automated Anatomical Labeling (AAL, version 1 for SPM8) atlas^37^ which consists of 116 cortical, subcortical and cerebellar regions of interest (ROIs, see Supplementary Table 1). The edges between the nodes were defined as the probability of fibre tracts or streamlines connecting the nodes. Structural probabilistic connectivity maps were generated using a modified Functional Magnetic Resonance Imaging of the Brain (FMRIB) Software Library (FSL, version 5.0.7) probabilistic fibre-tracking algorithm.^38,39^ Briefly, the diffusion properties were first calculated using the FSL DTI Toolkit, which included motion and eddy current correction. Bayesian estimation of diffusion parameters was then performed (bedpostx: number of fibres = 2, burnin period = 1000, jumps = 1250, multiexponential model, Rician noise). To create the seeds for fibre tracking, the diffusion data was registered to the 3DT1WI using FMRIB's Linear Image Registration Tool (FLIRT).^40,41^ The 3DT1WI was co-registered to the MNI 152 T1 2 mm brain (same coordinate system as the AAL atlas) using symmetric diffeomorphic image registration (Advanced Normalization Tools 2.4.4, transform type included rigid, affine and deformable symmetric image normalization).^42^ The transformations from diffusion to 3DT1WI and 3DT1WI to MNI space were concatenated to create transformations from diffusion to MNI space and their inverse and used to perform probabilistic fibre tracking (probtrackx2, 5000 samples, 2000 steps/sample, curvature threshold = 0.2) for each voxel within an AAL ROI. The streamline density maps were normalized by the number of samples (*n* = 5000) to generate the probability that a streamline from a seed voxel reached a voxel-of-interest. The union of these probability maps for all seeds within an AAL ROI was calculated to generate the probability that a streamline from any voxel in the AAL ROI reached a voxel-of-interest. This process was repeated to generate probability maps for all 116 ROIs. The weighted structural connectivity probability, *p_ij_*, between ROI *i* and ROI *j* was measured as the mean of the probability values within ROI *j* in the streamline probability map for ROI *i*. This resulted in 116 × 116 connectivity matrices with the elements representing the weighted structural connectivity probability, *p_ij_*. Connectivity matrices were calculated for individual patients and healthy controls (see Fig. 1A).

### Threshold analysis

To calculate network topological measures, a threshold was applied to eliminate spurious connections with low probabilities and ensure the association matrices retained small-world characteristics. We utilized absolute thresholds (setting weights below a target value to zero) to generate the association matrices.

Proportional thresholding (setting weights to zero to achieve a target network density) biases weak connections in less strongly connected networks, potentially retaining spurious connections and obscuring disease related disconnections.^43-45^ However, because global topological properties can be influenced by density,^45^ analysis was repeated using proportional thresholding.

### Network parameters

Four global topological network properties related to complex network efficiency were examined: clustering coefficient, global efficiency, average degree and hub index.^46^ All connectivity matrices were treated as binarized directed graphs in the computation of the network parameters using the Brain Connectivity Toolbox (BCT, version 2019)^47^ running in Matlab (Mathworks, R2023a) by an experimenter blinded to the patient outcomes. The BCT functions were used for calculating binarized directed clustering coefficient (clustering_coef_bd), global efficiency (efficiency_bin, with the argument ‘1’ to compute global efficiency) and degree (degree_dir). Matrices were thresholded and binarized with BCT’s threshold_absolute and weight_conversion functions. Proportional thresholding analysis used weighted and directed clustering coefficient (clustering_coef_wd), global efficiency (efficiency_wei) and strength (strength_dir) and matrices were thresholded using the threshold_proportional function.

#### Clustering coefficient

This network parameter quantifies the fraction of connected triangles surrounding each node, representing the proportion of a node’s neighbours that are also connected (Fig. 1B).^30^ It reflects local efficiency, where regions with high clustering efficiently relay information between nodes, indicating an anatomical organization that supports functional segregation within the network.

#### Global efficiency

Global efficiency quantifies the inverse of the average shortest path length (Fig. 1B) between all pairs of nodes within a network.^30^ It serves as a measure of the network’s global integration.^47^

#### Degree

The degree for a binarized network is the number of connections to and from a node. The global degree (i.e. average of the degree across all nodes in a network) reflects the network’s overall density or ‘wiring cost’.^47^

#### Strength

The strength of a node is the sum of the weighted edges to and from a node and is considered the weighted version of degree.^47^ The global strength is the average of the strength across all nodes in a network. This was used in place of degree for proportional thresholding analysis since degree is held constant.

#### Hub index

**Figure 2:**
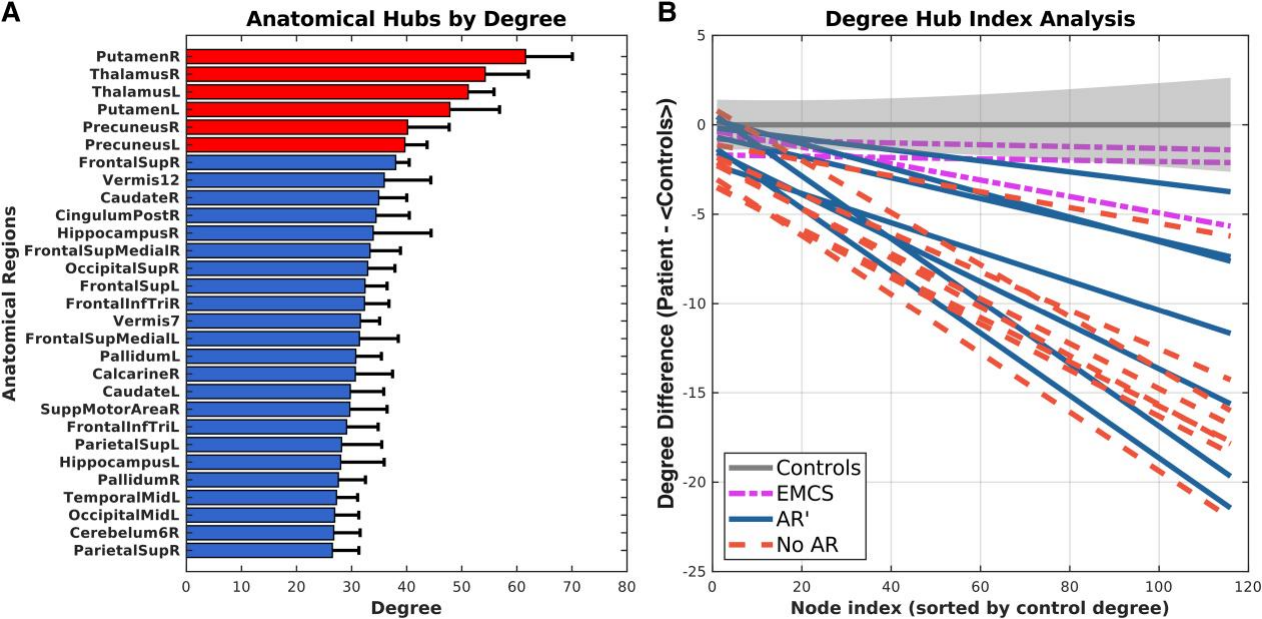
**Hub index analysis. (A)** Visualization of the top 25% of nodes identified by the average degree and standard deviation across all Control datapoints (*n* = 12). Supplementary Fig. 2 shows all the nodes. Definitions of anatomical regions are in Supplementary Table 1. The top 5% of hubs are highlighted in red. **(B)** Visualization of the hub index calculated as the slope of the lines. The lines of the best fit for points corresponding to the difference between individual nodal degree and average nodal degree of Control datapoints (*n* = 12) are shown. Each line represents the fitted line for one patient colour coded by group (EMCS [*n* = 3], AR′ [*n* = 7], No AR [*n* = 8]). AR': Arousal recovery patients who did not emerge from a minimally conscious state; CalcarineR: right calcarine cortex; CaudateL: left caudate; CaudateR: right caudate nucleus; Cerebelum6R: right lobule VI of the cerebellum; CingulumPostR: right posterior cingulum; EMCS: Arousal recovery patients who emerged from a minimally conscious state; FrontalInfTriL: left triangular part of the inferior frontal gyrus; FrontalInfTriR: right triangular part of the inferior frontal gyrus; FrontalSupL: left frontal superior gyrus; FrontalSupMedialL: left medial superior frontal cortex; FrontalSupMedialR: right medial superior frontal cortex; FrontalSupR: right frontal superior gyrus; HippocampusL: left hippocampus; HippocampusR: right hippocampus; No AR: No arousal recovery; OccipitalMidL: left middle occipital gyrus; OccipitalSupR: right superior occipital; PallidumL: left pallidum; PallidumR: right pallidum; ParietalSupL: left lobule of the superior parietal; ParietalSupR: right lobule of the superior parietal; PrecuneousL: left precuneous; PrecuneusR: right precuneous; PutamenL: left putamen; PutamenR: right putamen; SuppMotorAreaR: right supplementary motor area; TemporalMidL: left middle temporal gyrus; ThalamusL: left thalamus; ThalamusR: right thalamus; Vermis12: lobules I/II of the vermis; Vermis7: lobule VII of the vermis.

High-degree nodes are characteristic of essential hub areas.^48,49^ The hub index is a global measure developed by Achard *et al.*^46^ that quantifies how much high-degree nodes are altered compared with low-degree nodes in comatose patients relative to healthy controls. It was calculated by averaging the nodal degree across the twelve connectivity matrices from the four healthy controls and assigning node indices in ascending order according to their average degree (Fig. 2A, Supplementary Fig. 2 shows all the nodes). The highest node value (i.e. 116) was assigned to the node with the greatest average global degree and the lowest node value (1) was assigned to the node with the smallest average global degree (Fig. 2B). The difference between the degree for each participant's node and the corresponding average nodal degree from the Control cohort was calculated. A linear model was fitted using the node index as the regressor and the degree differences as the dependent variable, with the resulting slope used as the global hub index for the participant. This process was repeated for all patients. A negative slope would indicate that high-degree hubs have disproportionately reduced degrees compared with low-degree hubs. For proportional thresholding analysis, degree was replaced with strength. Similarly, a negative slope would suggest that high-strength hubs experienced a disproportionately greater reduction in strength than low-strength hubs.

### Threshold determination

A key feature of complex networks is small-world behaviour.^30,35,50^ Small-world networks bridge regular and random topologies, combining the high clustering of regular networks with the high global efficiency of random networks,^30^ enabling both local specialization and efficient global communication. The absolute and proportional thresholds were selected as the values which maintained small-world properties for global efficiency and clustering coefficient in the Control cohort. The threshold that resulted in the Control networks yielding clustering coefficients and global efficiency values between those of their matched random and regular networks was chosen (Supplementary Fig. 3).

To evaluate clustering and global efficiency in our patient and Control cohorts, random and regular networks were generated for each real participant network using BCT. Random networks generated using degree-preserving rewiring algorithms (randmio_dir) served as null models for global efficiency, reflecting the reduced clustering typical of random topologies.^50^ Degree-matched regular lattice networks (latmio_dir) served as null models for clustering coefficient, given their inherent high clustering and long path lengths (low global efficiency). Random and lattice null networks were calculated using 1000 iterations across absolute and proportional thresholds, ranging from zero to one in 0.01 intervals, matching the real networks’ in- and out-degree distribution. When the resultant thresholded matrix consisted of a single element, the thresholded matrix was used. The clustering coefficient and global efficiency were calculated for each threshold for all participants. The average for each group was calculated and compared. The average of the clustering coefficient and the global efficiency of the Controls were plotted along with the standard deviation to select the optimal threshold.

### Structural connectome visualization

BrainNet Viewer (version 1.7)^51^ was utilized to visualize network differences in key hubs across the Control, AR and No AR cohorts.^51^ The association matrices were averaged and thresholded to generate the edge values for each group. Absolute threshold analysis has binarized inputs (edges are the same size) while proportional threshold analysis utilized weighted edges. The coordinates of the nodes were based on the centroid of the AAL ROIs. The node sizes represented the average degree (absolute thresholding) and strength (proportional thresholding) across group participants.

### Statistical analysis

Statistical analyses comparing baseline patient characteristics were performed using R version 4.3.1 (The R Foundation for Statistical Computing). Univariable analyses were performed for categorical variables (Fisher’s Exact Test) and for continuous variables (Wilcoxon Rank Sum two-sample test with continuity approximation for two groups).^52^ Differences in network parameters between groups (short-term: Controls, AR, patients without arousal recovery [No AR] ; long-term: Controls, EMCS, AR′ [AR patients who do not emerge from the minimally conscious state] and No AR) were tested with the Kruskal–Wallis rank-sum test followed by post-hoc two-sample Wilcoxon Rank-Sum tests (approximate method). Kendall’s Tau test was used to identify trends related to short-term recovery (ordered: Controls, AR, no AR) and long-term outcomes (ordered: Controls, EMCS, AR′, No AR). For group comparisons, the mean network parameters across the three time points for the four Controls were used. Boxplots were generated using Matlab. The significance level was set to *P* < 0.05. Key hubs were defined as nodes with the top 5% degree values. Inkscape version 1.2 (The Inkscape Project) and Microsoft Powerpoint version 16.101.2 were used for image composition.

## Results

### Demographics and clinical characteristics

Twenty-two patients and five controls were enrolled. However, three patients did not undergo MRI exams (two patients had WLST prior to MRI, and one was unable to lie supine for an extended period). Additionally, one patient and one control participant were excluded because the number diffusion MRI slices acquired differed from that of the other participants. Thus, the final cohorts comprised 18 cardiac arrest patients and four healthy controls (2F/2M, age 40 ± 20 years). Demographic, clinical baseline characteristics and outcomes of the 18 patients are detailed in Table 1. MRI was performed primarily for clinical indications, with research sequences added to the protocol, except for five patients, for whom the MRI was performed primarily for research purposes. For these five patients, all in the AR group, an earlier clinical MRI had been acquired prior to enrolment. The median (IQR) time-to-MRI for primarily research MRI patients was 9 (8, 14) days, whereas the time-to-MRI for the primarily clinical MRI AR patients was 5 (4, 6) days (*P* = 0.027). Six of the 18 patients in our final cohort had been enrolled prior to the protocol amendment expanding follow-up visits up to 1 year (see Supplementary Methods). Of these six patients, one survived to discharge, and the 6-month and 1-year CPC and mRS scores were carried forward from the 3-month visit (CPC = 1, mRS = 0). Four of the 12 remaining patients (enrolled with planned 6-months and 1-year telephone follow-up visits) survived and completed all visits. Across the five survivors, three achieved EMCS, while two remained in a minimally conscious state.^53^ Two of the three EMCS patients had been scanned for primarily research purposes.

**Table 1:** Patient demographics and baseline characteristics, comparing patients with (AR) and without (no AR) arousal recovery prior to discharge

	Patients (*n* = 18)	AR (*n* = 10)	No AR (*n* = 8)	*P*-value
Age, years (mean ± SD)	50 ± 22	48 ± 26	53 ± 17	0.59
Sex, male (%)	8 (44%)	2 (20%)	6 (75%)	0.054
Primary cardiac aetiology, yes (%)	8 (44%)	3 (30%)	5 (63%)	0.34
Witnessed, yes (%)	9 (50%)	4 (40%)	5 (63%)	0.64
Non-shockable rhythm, yes (%)	10/17 (59%)	4/9 (44%)	6/8 (75%)	0.33
Hospital transfer, yes (%)	10 (56%)	6 (60%)	4 (50%)	1.00
TTM, yes (%)	17 (94%)	9 (90%)	8 (100%)	1.00
33°C	16/17 (94%)	8/9 (89%)	8/8 (100.0%)	1.00
36°C	1/17 (5.9%)	1/9 (11%)	0/8 (0.0%)	
Admission GCS, median (IQR)	3 (3, 4)	3 (3, 4)	3.00 (3.00, 3.50)	0.63
Admission pupillary light reflex, present (%)	7 (39%)	5 (50%)	2 (25%)	0.37
Admission corneal reflex, present (%)	6 (33%)	3 (30%)	3 (38%)	1.00
Admission cough reflex, present (%)	7/17 (41%)	3/9 (33%)	4 (50%)	0.64
**Imaging**				
Time-to-MRI, days, median (IQR)	5 (4, 6.75)	6.50 (5.25, 8.75)	4 (3, 5)	**0**.**010**
GCS on day of MRI, median (IQR)	4 (3, 5.75)	5.50 (4.25, 8)	3 (3, 3.25)	**0**.**002**
CRS-R on day of MRI, median (IQR)	1 (0, 3) (*n* = 17)	2.50 (1.25, 3) (*n* = 10)	0 (0, 0) (*n* = 7)	**<0**.**001**
Sedation at MRI, yes (%)	10 (56%)	3 (30%)	7 (88%)	**0**.**025**
Intubated or tracheostomized at MRI, yes (%)	14 (78%)	6 (60%)	8 (100%)	0.092
Research only MRI, yes (%)	5 (28%)	5 (50%)	0 (0%)	**0**.**036**
**Outcomes**				
In-hospital death, yes (%)	13 (72%)	5 (50%)	8 (100%)	**0**.**036**
Cause of death				1.00
Brain death, yes (%)	1/13 (8%)	0/5 (0%)	1/8 (13%)	
WLST, yes (%)	12/13 (92%)	5/5 (100%)	7/8 (88%)	
Time-to-death, days, median (IQR)	7 (6, 12) (*n* = 13)	9 (8, 16) (*n* = 5)	6 (5.75, 8.25) (*n* = 8)	0.055
EMCS, yes (%)	3 (17%)	3 (30%)	0 (0%)	0.22
Discharge CPC, median (IQR)	5 (4.25, 5)	4.50 (3.25, 5)	5 (5–5)	**0**.**028**
Discharge mRS, median (IQR)	6 (5.25, 6)	5.50 (5.00, 6)	6 (6–6)	**0**.**028**
90-day CPC, median (IQR)	5 (4.25, 5)	4.50 (3.00, 5)	5 (5–5)	**0**.**028**
90-day mRS, median (IQR)	6 (5.25, 6)	5.50 (4.25, 6)	6 (6, 6)	**0**.**028**
6-month CPC, median (IQR)	5 (3.50, 5)	4 (2.25, 5)	5 (5, 5)	**0**.**028**
6-month mRS, median (IQR)	6 (5.25, 6)	5.50 (4.25, 6)	6 (6, 6)	**0**.**028**
12-month CPC, median (IQR)	5 (3.50, 5)	4 (2.25, 5)	5 (5, 5)	**0**.**028**
12-month mRS, median (IQR)	6 (5.25, 6)	5.50 (4.25, 6)	6 (6, 6)	**0**.**028**

AR = Arousal Recovery; TTM = Targeted Temperature Management; GCS: Glasgow Coma Scale; CPC = Cerebral Performance Category; CRS-R: Coma Recovery Scale Revised; EMCS: Emergence from a Minimally Conscious State measured on the JFK Coma Recovery Scale—Revised test; WLST: Withdrawal of Life Sustaining Treatment; mRS: Modified Rankin Scale.

*P* < 0.05 in bold.

### Absolute threshold determination

Supplementary Fig. 3 illustrates the clustering coefficient and the global efficiency, averaged for the 12 healthy control scans and the 18 cardiac arrest patients across absolute thresholds from 0% to 100% probability. Both patient and control networks exhibited small-world properties across multiple thresholds, though at very high thresholds (i.e. >0.5) the networks became overly sparse resulting in low clustering and low efficiency networks. The patient group demonstrated qualitatively reduced clustering (indicating lower local efficiency) and reduced global efficiency compared with Controls. An absolute threshold of approximately 0.10 marked the point at which Control networks diverged from regular networks, suggesting a transition to greater structural complexity. This threshold was applied to all networks for the primary analysis.

### Association of arousal recovery with structural connectivity

**Figure 3:**
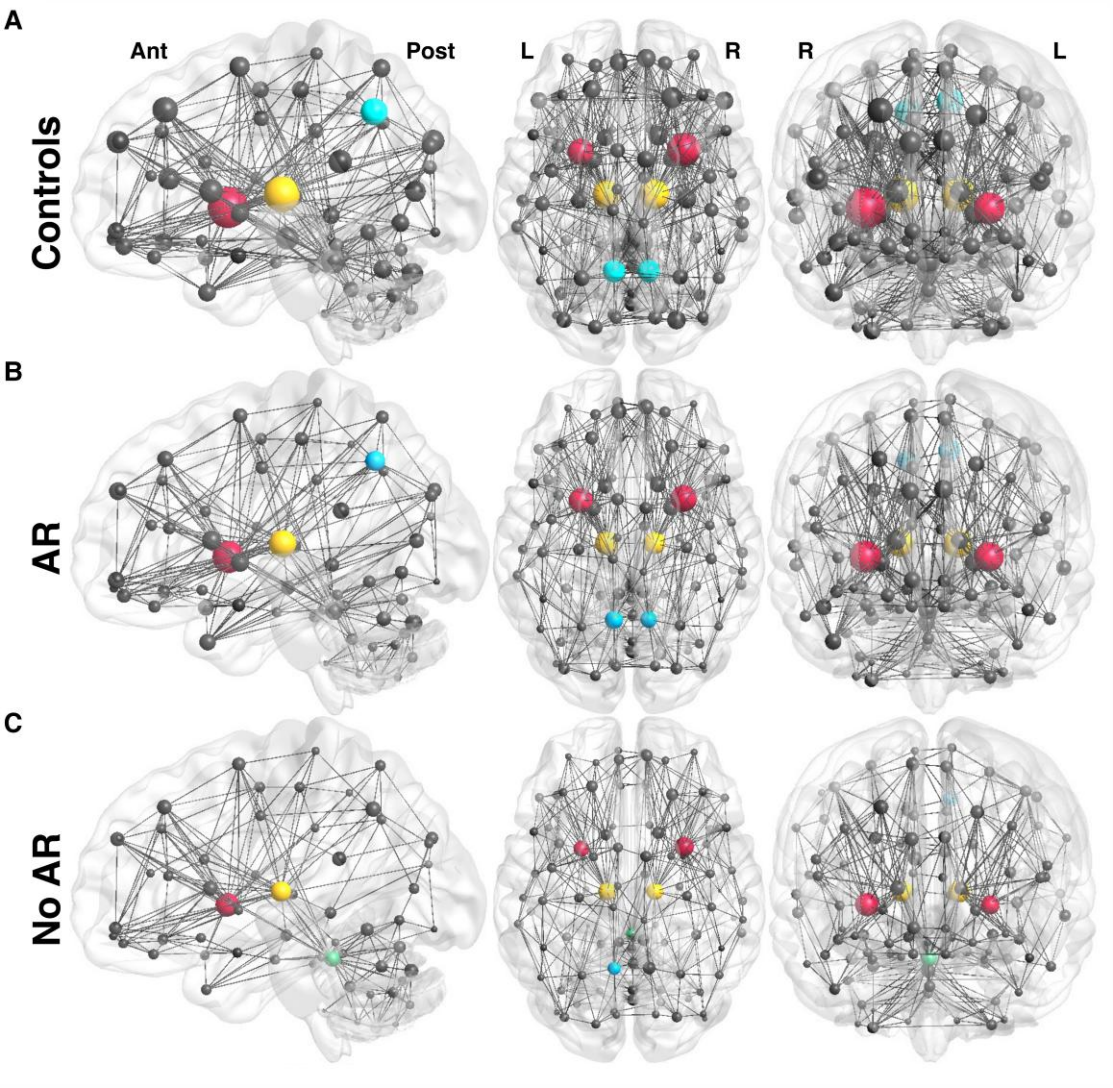
**Visualization of the average structural connectome for (A) controls (*n* = 12), (B) AR patients (*n* = 10) and (C) no AR patients (*n* = 8).** The top 5% of hubs (using an absolute threshold of 0.1) are shown (red—putamen, yellow—thalamus, blue—precuneus, green—vermis lobules I/II). The nodes’ size scales with their degree. Ant: Anterior; AR: Arousal recovery; L: Left; No AR: No arousal recovery; Post: Posterior; R: Right.

Examples of raw connectivity (Fig. 1A) and binarized matrices are shown (Fig. 1B) for a Control participant, an AR patient and a No AR patient. While the average structural connectome for Controls and patients with AR (Fig. 3) exhibit similar key hubs (Supplementary Table 2), the density of connections appears reduced for both the AR and No AR cohorts. Furthermore, the key hubs (top 6 nodes) for the average No AR cohort no longer included the right precuneus. Hub index analyses show a clear downward trend for all patients, signifying that high-degree nodes in healthy brains were disproportionally damaged across all patients compared with low-degree nodes (Fig. 2B).

**Figure 4:**
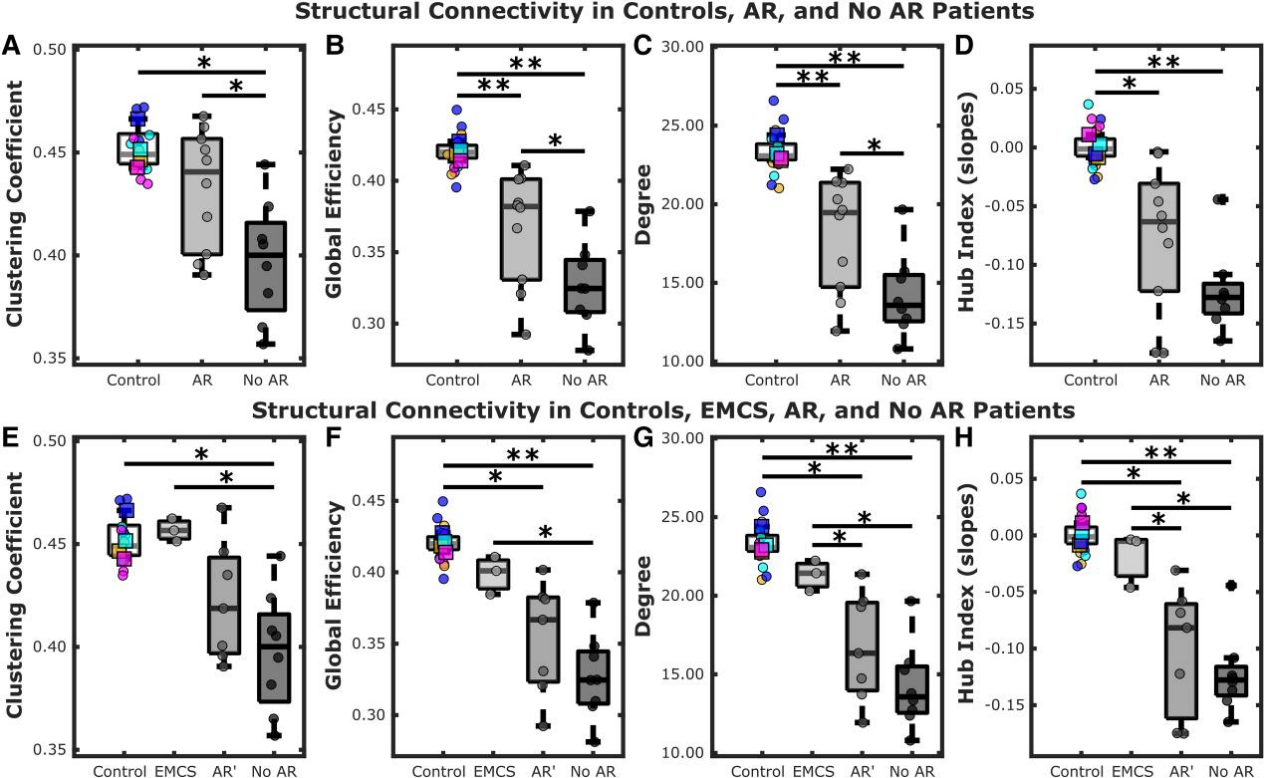
**Group-level differences in graph theory measures.** Differences in the graph theory measures for (**A**) clustering coefficient, **(B)** global efficiency, **(C)** degree and **(D)** hub index across Controls (*n* = 4), AR patients (*n* = 10) and No AR (*n* = 8) patients. Results separating AR patients who achieved EMCS (*n* = 3) from patients who did not (AR′) (*n* = 7) for **(E)** clustering coefficient, **(F)** global efficiency, **(G)** degree and **(H)** hub index. Statistical comparisons were performed using the Kruskal–Wallis rank-sum test (χ^2^ reported) followed by post-hoc two-sample Wilcoxon rank-sum tests (approximate method). χ^2^ and *P*-values for each comparison are provided in Tables 2 and 3.*P < 0.05. **P < 0.01. Individual participant data are shown as filled circles. The means of three repeated measures for the four Control participants are shown as squares. The Control data are colour coded by participant (magenta, orange, cyan, blue). AR: Arousal recovery; AR': Arousal recovery patients who did not emerge from a minimally conscious state; EMCS: Emergence from a minimally conscious state; No AR: No arousal recovery.

The clustering coefficient, global efficiency, degree and hub index of Controls, AR and No AR cohorts were compared (Fig. 4A–D, Table 2). Significant differences were observed between the three groups for all four metrics (Kruskal–Wallis Tests, *P* < 0.05). Kendall's Tau showed strong negative trends for all metrics (*P* < 0.01), meaning greater disorders of consciousness were associated with decreasing complexity values. Post-hoc Wilcoxon rank-sum testing showed that the Control group's values were significantly greater than No AR for all metrics. The Control group's values were significantly greater than AR for all metrics, except for the clustering coefficient. The AR group's values were significantly greater than the No AR cohort's values for all metrics, except for the hub index.

**Table 2:** Comparison of absolute thresholding network connectivity metrics between healthy control participants (controls), patients with arousal recovery (AR), and patients with no arousal recovery (No AR)

	Clustering coefficient	Global efficiency	Degree	Degree hub index
**Kruskal–Wallis Test**	**χ^2^(2)** **=** **8.06, *P* = 0.018**	**χ^2^(2)** **=** **12.46, *P* = 0.002**	**χ^2^(2)** **=** **12.21, *P* = 0.002**	**χ^2^(2)** **=** **9.51, *P* = 0.009**
**Controls versus AR** **(*P*-value)**	0.36	**0**.**006**	**0**.**006**	**0**.**028**
**Controls versus No AR** **(*P*-value)**	**0.014**	**0.008**	**0.008**	**0.008**
**AR versus No AR** **(*P*-value)**	**0.037**	**0.037**	**0.046**	0.12
**Kendall's Tau**	**τ** **=** **−0.49, *P* = 0.005**	**τ = −0.64, *P* < 0.001**	**τ = −0.63, *P* < 0.001**	**τ = −0.53, *P* = 0.002**

AR = arousal recovery; No AR = no arousal recovery.

*P* < 0.05 in bold.

**Table 3:** Comparison of absolute thresholding network connectivity metrics between healthy control participants (controls), patients with emergence from the minimally conscious state (EMCS), patients with arousal recovery who did not achieve EMCS (AR′), and patients with no arousal recovery (No AR)

	Clustering coefficient	Global efficiency	Degree	Degree hub index
**Kruskal–Wallis Test**	**χ^2^(3)** **=** **11.10, *P* = 0.011**	**χ^2^(3)** **=** **14.46, *P* = 0.002**	**χ^2^(3)** **=** **14.62, *P* = 0.002**	**χ^2^(3)** **=** **13.13, *P* = 0.004**
**Controls versus EMCS** **(*P*-value)**	0.60	0.052	0.052	0.60
**Controls versus AR′** **(*P*-value)**	0.11	**0**.**011**	**0**.**011**	**0**.**011**
**Controls versus No AR** **(*P*-value)**	**0**.**014**	**0**.**008**	**0**.**008**	**0**.**008**
**EMCS versus** AR′ **(*P*-value)**	0.11	0.068	**0**.**040**	**0**.**040**
**EMCS versus No AR** **(*P*-value)**	**0**.**019**	**0**.**019**	**0**.**019**	**0**.**032**
AR′ **versus No AR** **(*P*-value)**	0.18	0.18	0.22	0.45
**Kendall's Tau**	**τ** **=** **−0.54, *P* = 0.001**	**τ = −0.69, *P* = <0.001**	**τ = −0.69, *P* = <0.001**	**τ = −0.60, *P* = <0.001**

Results for controls versus No AR patients are the same as those reported in Table 2 and are shown here again for completeness.

AR = Arousal recovery; AR'=Patients with arousal recovery who did not emerge from the minimally conscious state; EMCS = Emergence from the minimally conscious state; No AR = No arousal recovery.

*P* < 0.05 in bold.

Subset analysis compared Controls, EMCS, AR′ and No AR cohorts across measures of clustering coefficient, global efficiency, degree and hub index (Fig. 4E–H, Table 3). There remained a significant difference between the four groups (Kruskal–Wallis Tests, *P* < 0.05), and Kendall's Tau continued to show strong negative trends (*P* ≤ 0.001) for all metrics. Post-hoc Wilcoxon rank-sum testing showed that Control values were greater than No AR for all metrics and AR′ except for the clustering coefficient. EMCS values were greater than the No AR values for all metrics. EMCS values were significantly different from AR′ values for degree and hub index, but not for the clustering coefficient and global efficiency. For all metrics, no statistically significant difference was found between AR′ and No AR cohorts.

### Proportional thresholding analysis

To assess the influence of comparing networks of different densities, we repeated network analysis using proportional (density) thresholding instead of absolute thresholding. Proportional thresholding retains a proportion of the strongest edges in a network to achieve a given density. Examples of a Control, AR and No AR subject network with absolute thresholding at 0.10 and proportional thresholding at 0.08 are visualized in Supplementary Fig. 4A and B, respectively. As can be seen qualitatively, the density of the graphs decreases from Control to No AR in the absolute thresholding case, whereas lower probability values are included in No AR compared with Controls in the proportional thresholding case to maintain density.

Supplementary Fig. 5 shows small-world analysis of networks for proportional thresholding. The average weighted and directed clustering coefficient and global efficiency were calculated for all groups as well as the corresponding regular and random graphs and plotted. The proportional threshold for which small-world properties were retained in Control networks was 0.08.

The methodology for proportional thresholding analysis was the same as for absolute thresholding except that the degree is replaced with strength. The hub index was computed using the strength of a node as an indication of its importance rather than its degree (Supplementary Figs 6A and 7). The top hubs were similar between proportional and absolute thresholding (i.e. thalamus, putamen) with the addition of lobules I/II and VII of the vermis as notable hubs instead of the left and right precuneus (Supplementary Fig. 8). Hub index analyses also show clear downward trends for all patients, signifying that high-degree nodes in healthy brains were disproportionally damaged across all patients compared with low-degree nodes (Supplementary Fig. 6B).

Structural connectivity changes using proportional thresholding for all four metrics are shown in Supplementary Fig. 9, Supplementary Tables 3 and 4 and are primarily consistent with the absolute thresholding results. Notably, in contrast to the absolute threshold results, a significant difference in the strength Hub Index was found between AR and No AR. Subset analysis between Controls, EMCS, AR′ and No AR cohorts also showed no significant difference between EMCS and AR′ for all four metrics, unlike the absolute threshold findings.

## Discussion

Our results show that structural connectivity measured using multi-shell diffusion imaging and graph network metrics can provide insight into DOC. Measuring disruptions to structural connectivity with graph network metrics showed that HIBI reduced brain network complexity for patients with poor outcomes. For all measures, DOC severity was significantly associated with loss of structural network complexity. Patients with worse outcomes exhibited more pronounced topological deviations from the Controls, while those with better outcomes demonstrated topological features more closely resembling Controls. Analysis of the AR and No AR groups generally revealed significant differences between the patient groups and Controls across all measures, indicating that HIBI perturbs the underlying topological complexity. However, the topological measures of EMCS patients aligned with those of the healthy Controls, while metrics from AR′ patients (i.e. AR patients without EMCS) were comparable to those from No AR patients. This suggests that AR without preserved structural network integrity may not lead to meaningful long-term recovery. Additional studies with larger sample sizes are needed to test this hypothesis. These findings support the possible utility of structural graph theory metrics for identifying patients with the greatest promise for recovering consciousness and most likely to benefit from aggressive interventions and avoidance of premature WLST.

### Structural changes in cardiac arrest patients and outcomes

Graph theory has revealed that small-world architecture is frequently disrupted in various neurologic and psychiatric disorders. Structural network analyses have identified altered small-world brain organization to various degrees in patients with schizophrenia,^54^ Alzheimer’s disease^55,56^ and traumatic brain injury.^57^ Clustering coefficient and global efficiency for the healthy Controls were measured against degree-matched random and lattice networks over a range of absolute thresholds and demonstrated a balance of local specialization (high clustering coefficient) and global efficiency (low characteristic path length) expected in complex brain networks,^30^ consistent with prior research.^27,28,58,59^ In contrast, patient networks exhibited considerable variability in both measures, likely reflecting the wide spectrum of brain damage severity, from EMCS to No AR. Qualitative analysis suggests that patient networks shift away from the segregation and global integration observed in Control networks (Supplementary Figs 3 and 5).^47^

In this study, the clustering coefficient was significantly reduced in the most severely impaired patients (No AR). Conversely, no significant differences in clustering coefficient were observed between Controls and any patient who achieved AR or better. This association between lower clustering coefficient and outcome severity suggests a diminished capacity for specialized and complex information processing in patients with worse outcomes, while those with better outcomes retain this capacity. Structural network studies in chronic DOC patients due to traumatic brain injury or HIBI produced conflicting clustering results. Weng *et al.*^59^ also found decreased clustering coefficient in patients, whereas Tan *et al*.^58^ reported increased clustering coefficients in patients compared with controls. However, Tan's study utilized deterministic fibre-tracking, and association matrices were based on the number of streamlines, whereas Weng's study employed probabilistic fibre-tracking and edges defined by probability of connection, a method similar to our implementation. Functional EEG studies also report increased clustering coefficient in early time-points (within 72 h)^60,61^ in patients with worse outcomes, however these studies might have been confounded by patients still receiving sedation during those times. However, another functional EEG study acquired in the chronic phase (mean time from injury was 3 years) also showed increased clustering coefficients related to poor outcomes.^62^ Nonetheless, the disparate time points of the EEG studies, with our study acquired in the subacute phase prior to discharge, and very different methodologies for generation of the association matrices make comparisons difficult. In contrast, a resting state fMRI study performed in the subacute stage also observed reduced clustering coefficient in patients with worse outcomes.^63^

Global efficiency effectively differentiated between Controls, patients who achieved AR or better and patients who did not achieve AR, with global efficiency decreasing with greater DOC severity. However, when EMCS patients were considered separately, their global efficiency did not significantly differ from Controls. This suggests that global integration is compromised in patients with poor outcomes but preserved in those who recover. Weng *et al*. found patients to have lower global efficiency than controls.^59^ Tan *et al.* found no significant difference in global efficiency between patients and controls, potentially due to the use of deterministic fibre-tracking.^58^

Degree was a key factor in distinguishing between cardiac arrest outcomes, decreasing progressively from Controls to patients who achieved AR and finally to those who did not. The lower degree in CA patients is due to the reduced probability of connections between AAL ROIs. This may be the result of widespread white matter tract damage, a known sequela of HIBI.^18,21,64^ Reduced connections have been noted in a functional EEG study of HIBI patients^60^ and in Weng *et al.*'s^59^ and Tan *et al.*'s^58^ studies.

### Hubs in cardiac arrest patients

This study found a correlation between patient outcome and the extent of decreased degree in hub nodes, as described by the hub index. This measure differentiated healthy controls from both patient groups. Furthermore, when EMCS was analysed separately, their hub indices were indistinguishable from those of Controls and significantly higher than patients who only achieved AR or had no AR. This suggests that patients with better outcomes retain connections in high-degree hubs, essential for global integration, whereas other patients have more severely impacted hub regions. This is supported by Crossley *et al.,*^48^ who showed that lesions are concentrated across hubs in brain disorders. This may be explained by the high metabolic demands of hubs,^65^ making them more susceptible to ischaemic injury.

FMRI studies in DOC patients have found that functional activity decreases in normal hub regions and increases in non-hub regions,^46,66,67^ aligning with this study’s findings. Underlying structural hub damage likely leads to decreased functional activity in hubs and compensatory increases in non-hub nodes. However, unlike functional hub reorganization, this study found no evidence of increases in the degree in lower-degree structural hubs among patients (Fig. 2B).

In our hub analysis, the top 5% of nodes by degree were identified as the bilateral thalami, putamen and precuneus regions in the healthy control networks (Fig. 2A, Fig. 4). Other regions maintaining high-degree hubs included the bilateral superior frontal gyrus, vermis lobules I/II, right caudate nucleus, right posterior cingulum, right hippocampus, right medial superior frontal cortex, right superior occipital gyrus and the triangular part of the right inferior frontal cortex. These findings align with anatomical hubs identified in prior studies, such as the superior frontal, precuneus, posterior cingulate, cerebellum, thalamus and putamen regions.^28,56,65^

Notably, the thalamus has been recognized as crucial for consciousness due to its functional deactivation under anesthesia^68^ and in patients with DOC,^22,69^ leading to impaired connection to the posterior cingulate and precuneal cortices.^70,71^ Similarly, the putamen is highly vulnerable to damage during cardiac arrest.^20,21^ These hub regions were disproportionately affected in patients with worse outcomes, further supporting their role in normal conscious processing.

The findings of this study align with theoretical frameworks of consciousness.^35,36^ Patients with worse outcomes exhibited disruptions in small-world properties suggesting both impaired local specialization (reduced clustering coefficient) and global integration (decreased global efficiency). Conversely, patients achieving EMCS demonstrated topological measures comparable to those of healthy Controls, indicating the preservation of network architecture is critical for integrating and broadcasting information. Furthermore, the hub index revealed the disproportionate vulnerability of high-degree hubs, such as the thalamus, precuneus, posterior cingulate cortex and frontal regions which are all known to be potential correlates of consciousness.^68,72^

### Strengths and limitations

A strength of this study is its use of multi-shell diffusion imaging which leverages multiple b-values to provide greater reliability in discerning crossing white matter tracts, thereby enabling more accurate tractography.^73^ The MRI sequence is available commercially across vendors with a clinically feasible acquisition time of less than 6 min. The integration of probabilistic tractography with graph theory to examine global structural changes may offer insights into the underlying neuropathology of DOC following CA. Previous studies focused on predefined fibre tracts,^74^ potentially missing global structural connectivity disruptions or on chronic time points months after the cardiac arrest.^58,59^ Furthermore, the approach presented here does not require manual delineation of regions of interests, making it suitable for routine clinical deployment. The time-consuming nature of probabilistic tractography calculation may be considered disadvantageous, but continuing computational power improvements will likely overcome this potential barrier to routine clinical implementation. Another strength of the proposed method is the provision of quantitative metrics of brain connectivity, i.e. clustering coefficient, global efficiency, global degree and hub index. By facilitating the identification of patients with preserved brain structural network topology using objective imaging biomarkers, these tools could greatly assist clinicians and families in managing expectations regarding potential outcomes and guide life-sustaining treatment decisions. However, additional studies will be needed to assess the reproducibility of these metrics using different scanners from multiple centres.

Some methodological issues should be considered when interpreting the results. Graph network results are sensitive to the choice of thresholds and thresholding method (e.g. absolute versus proportional). An absolute threshold approach was chosen due to the a priori hypothesis that connection probability would be reduced in HIBI patients and reflected in the degree metric. While comparing graph networks using absolute thresholding is challenging because global topological measures are sensitive to network density,^45^ equivalent results were achieved for clustering coefficient and global efficiency when using proportional thresholding (which compares networks of the same density). This consistency demonstrates that the topological alterations identified in patients with CA were robust to variations in thresholding strategy and persist even when network density is controlled.

The small sample size of this study may also reduce generalizability. There was heterogeneity in cardiac arrest causes, ranging from progressive heart failure to drug overdose. The No AR group had a higher rate of sedation during MRI acquisition and was scanned earlier post-arrest than the AR group, most likely due to the greater number of patients in the AR group who were imaged primarily for research purposes. Both sedation during MRI^75^ and timing of the scan after cardiac arrest^20^ have been shown to influence diffusivity measurements. Accordingly, we cannot exclude the possibility that sedation and time to MRI contributed to some of the observed group differences. The choice of parcellation scheme could have influenced results, as different atlases (e.g. Harvard Oxford Atlas-110 versus AAL-116) yield varying outcomes.^59^ Involuntary head movements during MRI scans may have introduced motion artefacts that could not be completely corrected. Spatial normalization of MR images, particularly for small ROIs, is challenging due to brain atrophy in cardiac arrest patients, potentially causing misalignment and variability of automated ROI seed specification.^65^ Using higher resolution diffusion MRI acquisitions, at the expense of longer acquisition times, may mitigate this confound. Additionally, there is no gold standard for structural MRI network construction, as variations in node definition, edge weights, diffusion MRI protocols and fibre tracking may lead to spurious or missed connections.^76^ Furthermore, exploring structural and functional networks together may provide a more comprehensive understanding of cardiac arrest's impact on the brain. However, functional network connectivity results are often potentially confounded by sedation in this patient population.^60,61,63^

Potential limitations posed by the patient cohort have been previously discussed.^32^ Notably, these include WLST which is common in this population.^77^ WLST can occur in patients where it is likely that they will be left with a disability, including cognitive, that would have been unacceptable to them according to their family or a living will or advance directive. Patients may also undergo WLST for non-neurological reasons, including poor systemic function (e.g. cardiac, renal). The study's patient population may have been skewed by the exclusion of patients with severe injury on admission CT as the primary reason for screen failure was WLST.^32^ Of the 13 patients who died in-hospital, only one was due to brain death and the rest was from WLST. For the ten AR patients, WLST occurred in five. The potential for additional recovery in these patients cannot be fully known, including achieving EMCS, if not for WLST. Importantly, structural connectivity metrics were not shared with the clinical team and thus did not influence WLST decisions. Additionally, patients had to be medically stable enough to undergo an MRI, which may also limit the generalizability of our findings.

## Conclusions

This research highlights the potential utility of multi-shell diffusion imaging and graph theory to assess structural brain network changes in comatose HIBI patients associated with the recovery of consciousness. The findings reveal that small-world network properties—critical for balancing local specialization and global integration—are disrupted to varying degrees and correlate with patient outcomes. The clustering coefficient, global efficiency and degree distinguished between patients with favourable and unfavourable outcomes, while the hub index underscored the vulnerability of high-degree nodes to ischaemic damage. These results suggest a role for structural network analysis in clinical practice in enhancing clinical neuroprognostication accuracy. Future studies with larger cohorts and combined functional and structural connectivity analysis may further refine these prognostic models and deepen our understanding of the mechanisms underlying consciousness recovery after hypoxic ischaemic brain injury.

## Supplementary Material

fcag117_Supplementary_Data

## Data Availability

The data that support the findings of this study are available on request from the corresponding author, pending approval of the local Institutional Review Board, with appropriate data usage agreement. The data are not publicly available due to privacy concerns of individuals that participated in the study. Code used for analyses are available at the Harvard Dataverse at https://doi.org/10.7910/DVN/PYJSYC.^78^
