# Supplementary material for: Connectome disruptions after hypoxic-ischaemic injury associate with consciousness disorder severity
Source: Brain Commun. 2026 Apr 2;8(2):fcag117. doi: 10.1093/braincomms/fcag117 (PMC13111490; doi:10.1093/braincomms/fcag117)
Supplement: fcag117_Supplementary_Data [file fcag117_supplementary_data.pdf]

Supplementary Material

Connectome disruptions after hypoxic-ischaemic injury associate with consciousness disorder severity

Sofia G. Hilger BA[1], Eric S. Rosenthal MD[2], Annelise M Kulpanowski DO[1], Jacob A. Dodelson BS[1], Gaston Cudemus-Deseda MD[3], Marjorie Villien PhD[1], Brian L. Edlow MD[1,2], James L. Januzzi MD[4], MingMing Ning MD[2], W. Taylor Kimberly MD PhD[2], Edilberto Amorim MD[2], M Brandon Westover MD PhD[5], William A. Copen MD[6], Pamela W. Schaefer MD[6], Joseph T. Giacino PhD[7], David M. Greer MD[8], Ona Wu PhD [1]

Author affiliations:

1 Athinoula A. Martinos Center for Biomedical Imaging, Department of Radiology, Massachusetts General Hospital, Harvard Medical School, Charlestown, MA 02129, USA

2 Department of Neurology, Massachusetts General Hospital, Boston, MA, 02114, USA

3 Department of Cardiac Anesthesiology and Critical Care Medicine, Massachusetts General Hospital, Boston, MA, 02114, USA

4 Department of Medicine, Cardiology Division, Massachusetts General Hospital and Baim Institute for Clinical Research, Boston, MA, 02114, USA

5 Beth Israel Deaconess Medical Center, Boston, MA 02215, USA

6 Department of Radiology, Neuroradiology Division, Massachusetts General Hospital, Boston, MA, 02114, USA

7 Department of Physical Medicine and Rehabilitation, Spaulding Rehabilitation Hospital, Harvard Medical School, Charlestown, MA 02129, USA

8 Department of Neurology, Boston University School of Medicine, Boston Medical Center, Boston, MA, 02118, USA

Correspondence to: Ona Wu, PhD

Athinoula A. Martinos Center for Biomedical Imaging
149 13th Street, CNY 2301
Charlestown, MA 02129
E-mail: ona.wu@mgh.harvard.edu

Supplementary Methods

Participants

Protocol changes for this study has been previously described in detail in the Supplement of Dhakal, Rosenthal et. al.[1] Initially, the study was limited to ten English-speaking witnessed out-of-hospital cardiac arrest patients who had a pre-arrest modified Rankin Scale (mRS) score of 0, were treated with targeted temperature management (TTM), and remained comatose 72 hours post-rewarming. Patients with initial absent pupillary or corneal reflexes were excluded, and follow-up was restricted to three months. The protocol was revised to include patients who were comatose at 24 hours post-rewarming, while still excluding those with absent brainstem reflexes, due to insufficient enrollment over the first eight months. The study was further expanded four months later to include patients with unwitnessed cardiac arrests, absent brainstem reflexes, with pre-arrest mRS scores greater than 0, and non-English speakers, as enrollment had continued to lag. Additional subsequent amendments included: (1) extending follow-up to include six-month and one-year telephone assessments, (2) allowing enrollment of patients not treated with TTM, (3) including in-hospital cardiac arrest patients, (4) adding a repeat MRI at two weeks, (5) removing the requirement that patients remain comatose for at least 24 hours, and (6) expanding enrollment to 22 patients. The enrollment flowchart is shown in Supplementary Fig. 1.

Supplementary Tables

Supplementary Table 1. Automated Anatomical Labeling (AAL) atlas[2] regions of interest (116) and the labels used in this study for which L and R represent left, and right respectively.

Left and Right Amygdala (AmygdalaL, AmygdalaR)
Left and Right Angular Gyrus (AngularL, AngularR)
Left and Right Calcarine Cortex (CalcarineL, CalcarineR)
Left and Right Caudate Nucleus (CaudateL, CaudateR)
Left and Right Anterior Cingulum (CingulumAntL, CingulumAntR), Left and Right Middle Cingulum (CingulumMidL, CingulumMidR), and Left and Right Posterior Cingulum (CingulumPostL, CingulumPostR)
Left and Right Cuneus (CuneusL, CuneusR)
Left and Right Triangular parts of the Inferior Frontal Gyrus (FrontalInfTriL, FrontalInfTriR), Left and Right Opercular parts of the Inferior Frontal Gyrus (FrontalInfOperL, FrontalInfOperR), and the Left and Right Orbital parts of the Inferior Frontal Gyrus (FrontalInfOrbL, FrontalInfOrbR)
Left and Right Medial Orbital Frontal Cortex (FrontalMedOrbL, FrontalMedOrbR), Left and Right Middle Orbital Frontal Cortex (FrontalMidOrbL, FrontalMidOrbR), and the Left and Right Superior Orbital Frontal Cortex (FrontalSupOrbL, FrontalSupOrbR)
Left and Right Superior Frontal Gyrus (FrontalSupL, FrontalSupR), and Left and Right Middle Frontal Gyrus (FrontalMidL, FrontalMidR)
Left and Right Medial Superior Frontal Cortex (FrontalSupMedialL, FrontalSupMedialR)
Left and Right Fusiform Gyrus (FusiformL, FusiformR)
Left and Right Heschl's Gyrus (HeschlL, HeschlR)
Left and Right Hippocampus (HippocampusL, HippocampusR)
Left and Right Insula (InsulaL, InsulaR)
Left and Right Lingual (LingualL, LingualR)
Left and Right Inferior Occipital Gyrus (OccipitalInfL, OccipitalInfR), Left and Right Middle Occipital Gyrus (OccipitalMidL, OccipitalMidR), and Left and Right Superior Occipital Gyrus (OccipitalSupL, OccipitalSupR)
Left and Right Olfactory Cortex (OlfactoryL, OlfactoryR)
Left and Right Pallidum (PallidumL, PallidumR)
Left and Right lobule of the Paracentral (ParacentralLobuleL, ParacentralLobuleR)
Left and Right Para Hippocampal Gyrus (ParaHippocampalL, ParaHippocampalR)
Left and Right lobule of the Inferior Parietal (ParietalInfL, ParietalInfR) and Left and Right lobule of the Superior Parietal (ParietalSupL, ParietalSupR)
Left and Right Postcentral Gyrus (PostcentralL, PostcentralR)
Left and Right Precentral Gyrus (PrecentralL, PrecentralR)
Left and Right Precuneus (PrecuneusL, PrecuneusR)
Left and Right Putamen (PutamenL, PutamenR)
Left and Right Gyrus Rectus (RectusL, RectusR)
Left and Right Rolandic Operculum (RolandicOperL, RolandicOperR)
Left and Right Supplementary Motor Area (SuppMotorAreaL, SuppMotorAreaR)
Left and Right Supramarginal Gyrus (SupraMarginalL, SupraMarginalR)
Left and Right Inferior Temporal Gyrus (TemporalInfL, TemporalInfR), Left and Right Middle Temporal Gyrus (TemporalMidL, TemporalMidR), and Left and Right Superior Temporal Gyrus (TemporalSupL, TemporalSupR)
Left and Right Middle Temporal Pole (TemporalPoleMidL, TemporalPoleMidR) and Left and Right Superior Temporal Pole (TemporalPoleSupL, TemporalPoleSupR)
Left and Right Thalamus (ThalamusL, ThalamusR)
Left and Right lobule III of the Cerebellum (Cerebelum3L, Cerebelum3R), Left and Right lobules IV and V of the Cerebellum (Cerebelum45L, Cerebelum45R), Left and Right lobule VI of the Cerebellum (Cerebelum6L, Cerebelum6R), Left and Right lobule VIIB of the Cerebllum (Cerebelum7bL, Cerebelum7bR), Left and Right lobule VIII of the Cerebellum (Cerebelum8L, Cerbelum8R), Left and Right lobule IX of the Cerebellum (, Cerebelum9L, Cerebelum9R), and Left and Right lobule X of the Cerebellum (Cerebelum10L, Cerebelum10R)
Left and Right Cerebellum Crus I (CerebelumCrus1L, CerebelumCrus1R) and Left and Right Cerebellum Crus II (CerebelumCrus2L, CerebelumCrus2R)
Lobules I / II of the Vermis (Vermis12), lobule III of the Vermis (Vermis 3), lobules IV and V of the Vermis (Vermis45), lobule VI of the Vermis (Vermis6), lobule VII of the Vermis (Vermis7), lobule VIII of the Vermis (Vermis8), lobule IX of the Vermis (Vermis9), lobule X of the Vermis (Vermis10)

Supplementary Table 2. Top 10 nodes by average degree for controls, arousal recovery (AR) patients and patients without arousal recovery (No AR). Definitions of anatomical regions are in Supplementary Table 1.

	Controls		AR		No AR	
	Label	Degree	Label	Degree	Label	Degree
**1.**	PutamenR	61.58	PutamenR	47.90	PutamenR	34.38
**2.**	ThalamusR	54.25	PutamenL	44.20	ThalamusL	31.50
**3.**	PutamenL	51.17	ThalamusL	42.50	ThalamusR	29.88
**4.**	ThalamusL	47.83	ThalamusR	40.10	PutamenL	27.25
**5.**	PrecuneusR	40.17	PrecuneusL	31.70	Vermis12	23.00
**6.**	PrecuneusL	39.67	PrecuneusR	29.00	PrecuneusL	22.25
**7.**	FrontSupR	38.00	CaudateR	28,00	PrecuneusR	21.88
**8.**	Vermis12	35.92	PallidumL	27.20	CaudateR	21.13
**9.**	CaudateR	34.92	FrontSupMedialL	27.00	FrontSupR	20.88
**10**	CingulumPostR	34.42	PallidumR	26.60	CingulumPostR	20.25

Supplementary Table 3. Comparison of proportional thresholding network connectivity metrics between healthy control participants (Controls), patients with arousal recovery (AR), and patients with no arousal recovery (No AR). Statisticaly significant findings (p<0.05) are in bold.

	Clustering Coefficient	Global Efficiency	Strength	Strength Hub Index
**Kruskal-Wallis Test**	**$\chi^2(2)=8.77$, $p=0.012$**	**$\chi^2(2)=10.63$, $p=0.005$**	**$\chi^2(2)=11.54$, $p=0.003$**	**$\chi^2(2)=12.53$, $p=0.002$**
**Controls vs AR (p-value)**	0.29	**0.040**	**0.028**	**0.013**
**Controls vs No AR (p-value)**	**0.008**	**0.008**	**0.008**	**0.008**
**AR vs No AR (p-value)**	**0.037**	**0.037**	**0.023**	**0.019**
**Kendall's Tau**	**τ =-0.51, $p=0.003$**	**$\tau=-0.59$, $p<0.001$**	**$\tau=-0.62$, $p<0.001$**	**$\tau=-0.65$, $p<0.001$**

Supplementary Table 4. Comparison of proportional thresholding network connectivity metrics between healthy control participants (Controls), patients with emergence from the minimally conscious state (EMCS), patients with arousal recovery who did not achieve EMCS (AR'), and patients with no arousal recovery (No AR). Results for controls vs. No AR patient are the same as those reported in Supplementary Table 3 and are shown here again for completeness. Statistically significant findings (p<0.05) are in bold.

	Clustering Coefficient	Global Efficiency	Strength	Strength Hub Index
**Kruskal-Wallis Test**	**$\chi^2(3)=11.55$, $p=0.009$**	**$\chi^2(3)=13.45$, $p=0.004$**	**$\chi^2(3)=13.91$, $p=0.003$**	**$\chi^2(3)=14.38$, $p=0.002$**
**Controls vs EMCS (p-value)**	0.86	0.60	0.38	0.22
**Controls vs AR' (p-value)**	0.11	**0.018**	**0.018**	**0.011**
**Controls vs No AR (p-value)**	**0.008**	**0.008**	**0.008**	**0.008**
**EMCS vs AR' (p-value)**	0.11	0.068	0.068	0.11
**EMCS vs No AR (p-value)**	**0.019**	**0.019**	**0.019**	**0.019**
**AR' vs No AR (p-value)**	0.18	0.18	0.12	0.09
**Kendall's Tau**	**τ =-0.56, $p<0.001$**	**$\tau=-0.64$, $p<0.001$**	**$\tau=-0.67$, $p<0.001$**	**$\tau=-0.69$, $p<0.001$**

Supplementary Figures

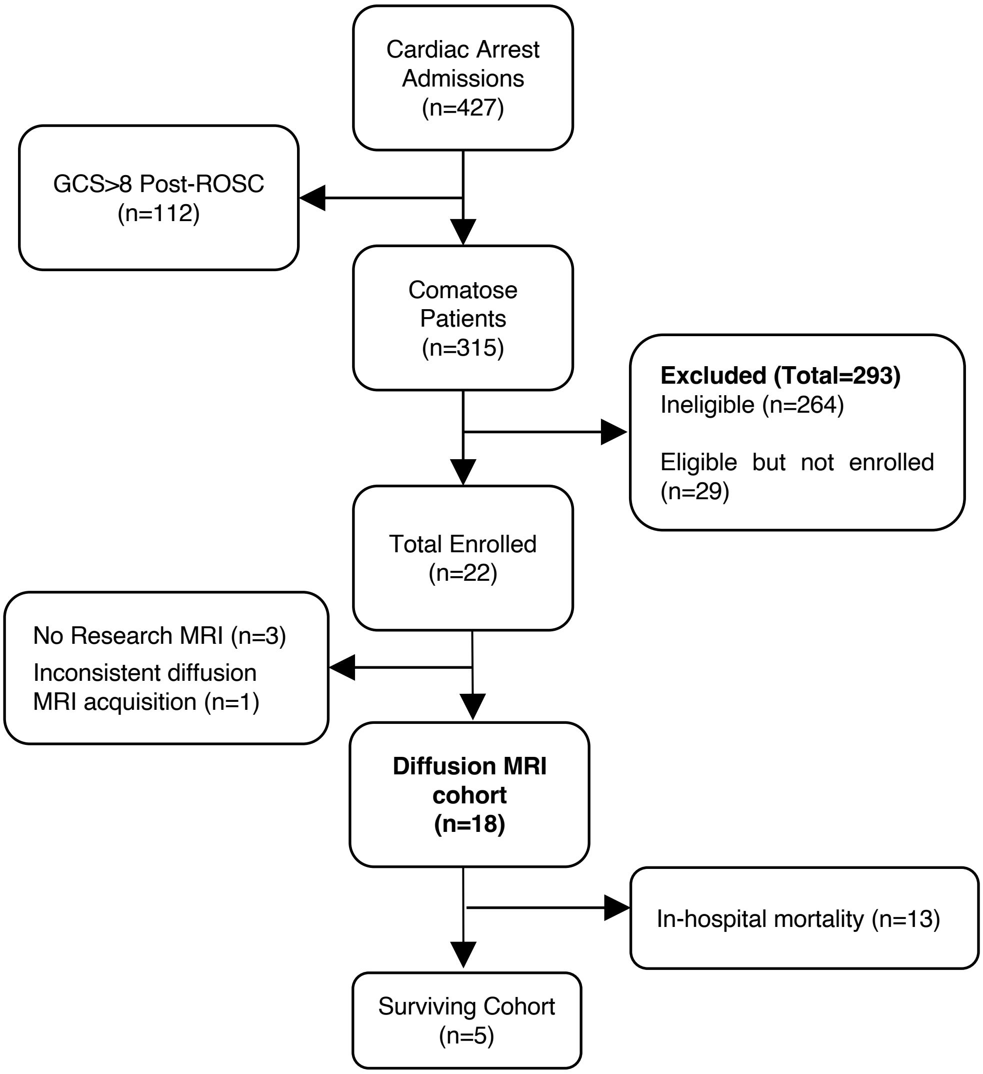

Supplementary Figure 1. A flowchart of patient screening, enrollment, and retention. Details for patient exclusion can be found in Dhakal, et al.[1] GCS: Glasgow coma scale; ROSC: return of spontaneous circulation.

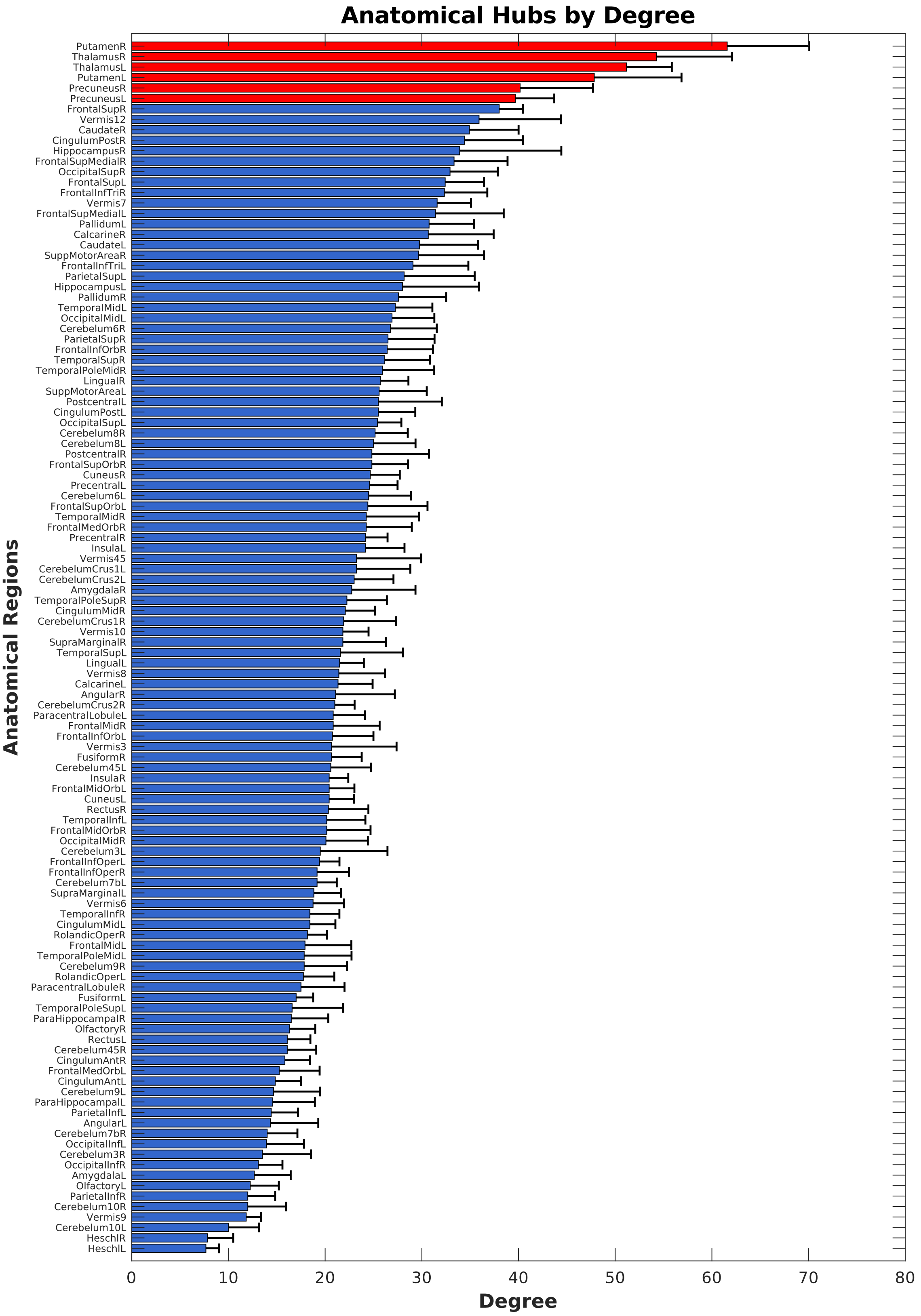

Supplementary Figure 2. Average degree and standard deviations of all 116 nodes in absolute threshold analysis. Nodes are ordered by decreasing average nodal degree across all Control datapoints (n=12). Definitions of anatomical regions are in Supplementary Table 1. The top 5% of hubs are depicted in red.

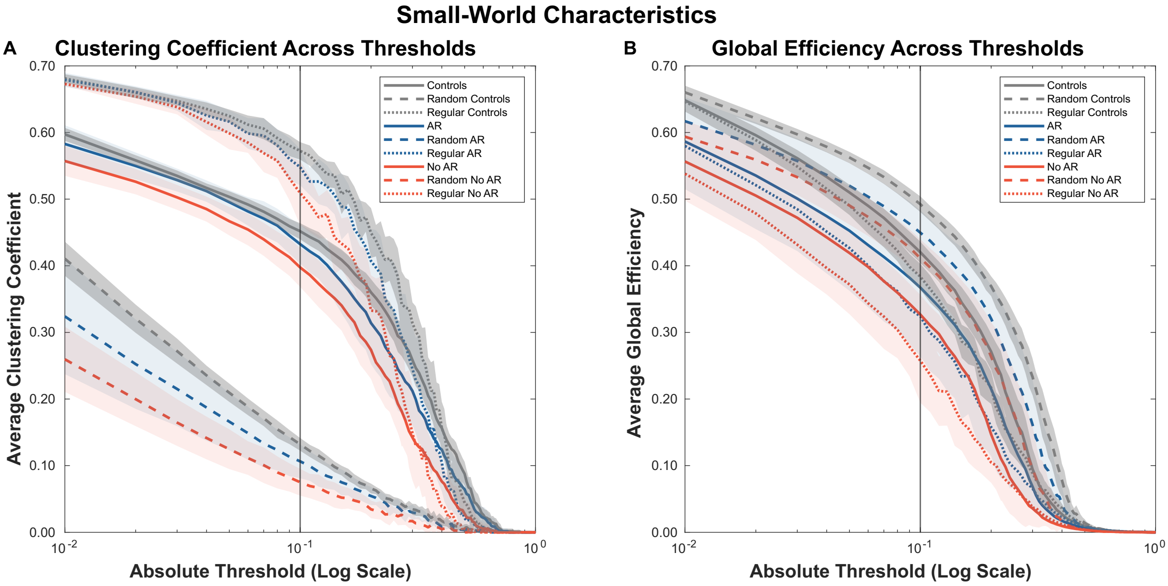

Supplementary Figure 3. Small-world characteristics of controls (n=12), AR (n=10) and No AR (n=8) patients compared to null random and regular networks. 100 absolute thresholds from zero to one were applied to the networks. Random and regular networks were generated using the functions randmio_dir and latmio_dir[3] matched to the degree of the input matrix. Matrices were binarised before analysis. The mean (line) and standard deviation (shaded regions) of the calculated (A) clustering coefficient and (B) global efficiency are shown on log x-scale. The vertical line represents the absolute threshold (0.10) used for the analysis. For all thresholds, the Controls exhibited greater clustering coefficients and global efficiency than both the AR and the No AR patients. Note that at the chosen threshold, the structural connectivity networks for the Controls, AR and No AR patients retained their small-world properties, i.e. lying between a random and regular network. AR: arousal recovery; No AR: No arousal recovery.

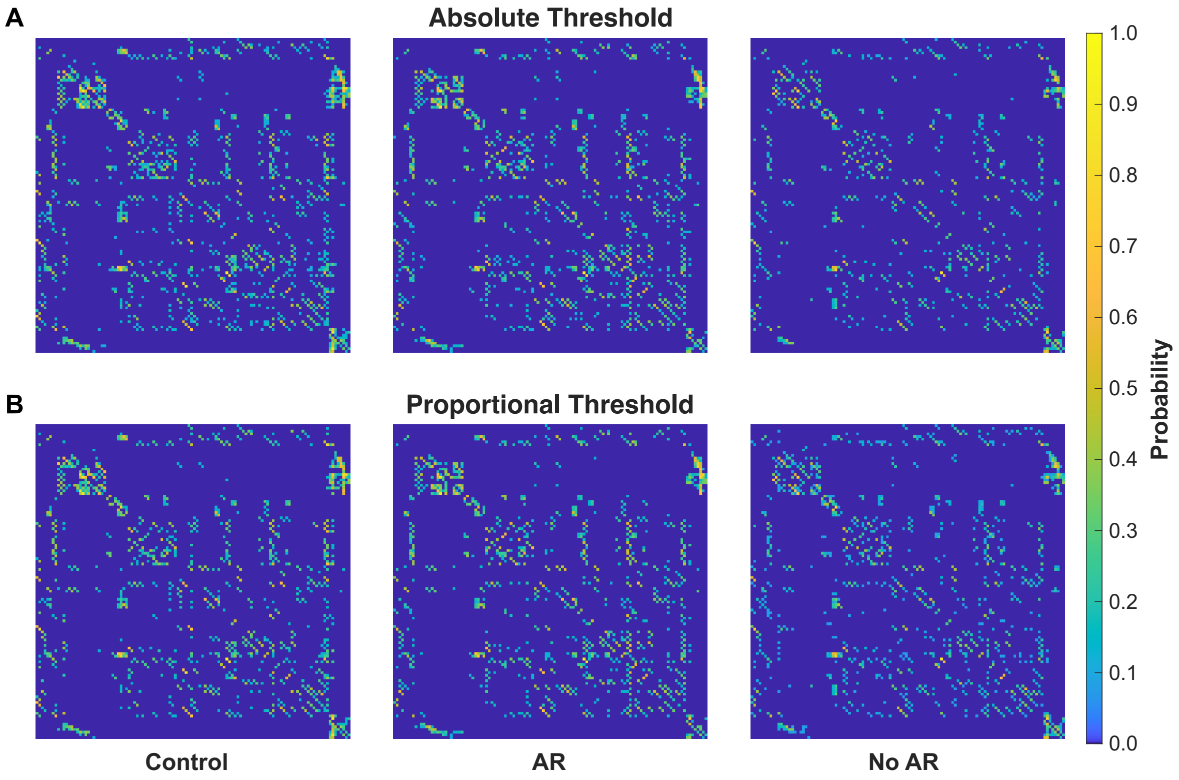

Supplementary Figure 4. Comparison of absolute and proportional thresholding. (A) An absolute threshold of 0.10 is applied to an example Control participant, AR and No AR patient networks. (B) A proportional threshold of 0.08 is applied to the same Control, AR and No AR participants shown in (A.) The probabilistic weight of each connection is expressed as a colour. AR: Arousal recovery; No AR: No arousal recovery.

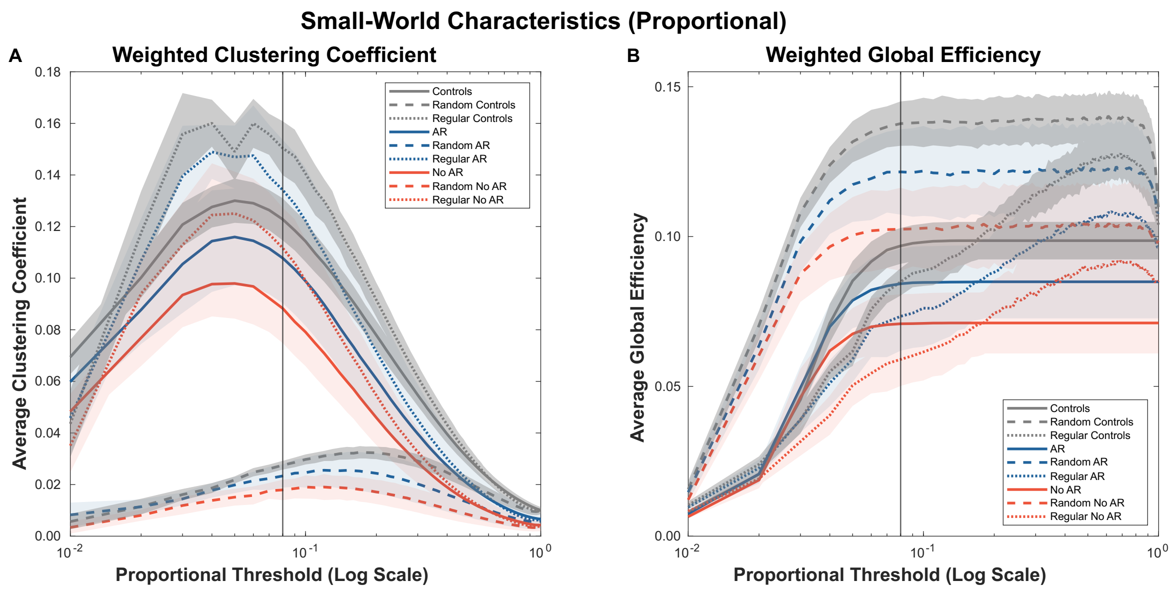

Supplementary Figure 5. Small-world characteristics of controls (n=12), AR (n=10), and No AR (n=8) patients compared to null random and regular networks across proportional thresholds. 100 proportional thresholds from zero to one were applied to the networks. Null networks were generated using the functions randmio_dir and latmio_dir,[3] modified to handle degenerate matrices. The mean (line) and standard deviation (shaded regions) of the calculated (A) clustering coefficient and (B) global efficiency are shown on a log x-scale. The vertical line represents the proportional threshold used for the rest of the analysis (0.08). For all thresholds, the Controls exhibited greater clustering coefficients and global efficiency than both the AR and the No AR patients. Note that at the chosen threshold, the structural connectivity networks for the Controls, AR and No AR patients retained their small-world properties, i.e. high clustering and high global efficiency. AR: arousal recovery; No AR: No arousal recovery.

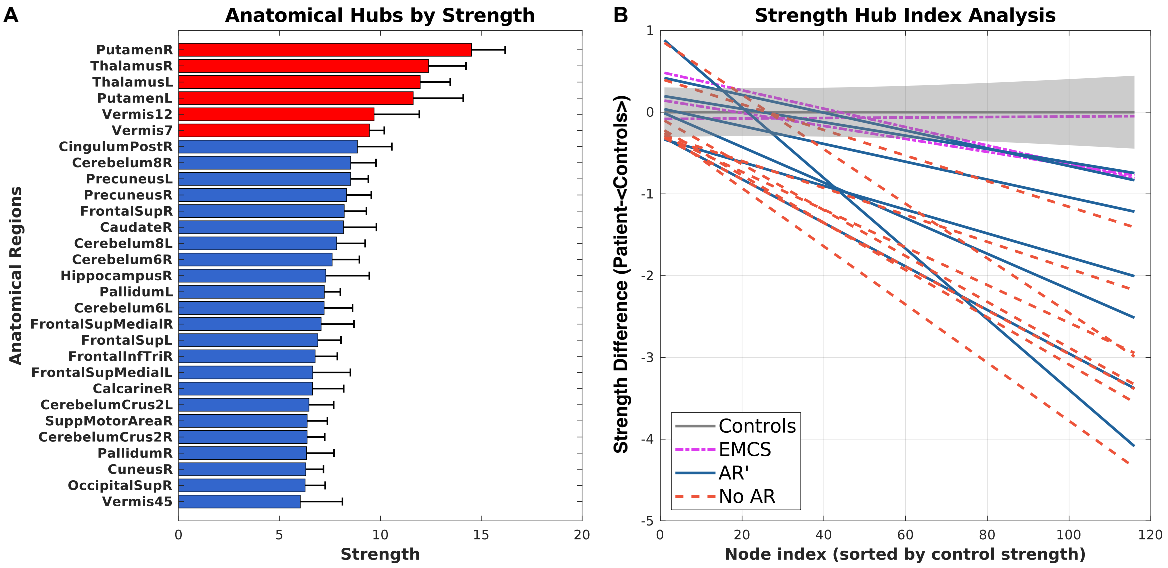

Supplementary Figure 6. Hub index analysis for strength analysis. (A) Visualisation of the top 25% of nodes identified by the average strength and standard deviation across all Control datapoints (n=12). Definitions of anatomical regions are in Supplementary Table 1. The top 5% of hubs are visualised in red. **(B)** Visualisation of the hub index calculated as the slope of the lines. The lines seen above are the lines of best fit for points corresponding to the difference between individual nodal strength and average nodal strength in controls. Each line represents the line of best fit for one patient colour coded by group (EMCS [n=3], AR' [n=7], No AR [n=8]). AR': Arousal recovery patients who did not emerge from a minimally conscious state; EMCS: Arousal recovery patients who emerged from a minimally conscious state; No AR: No arousal recovery.

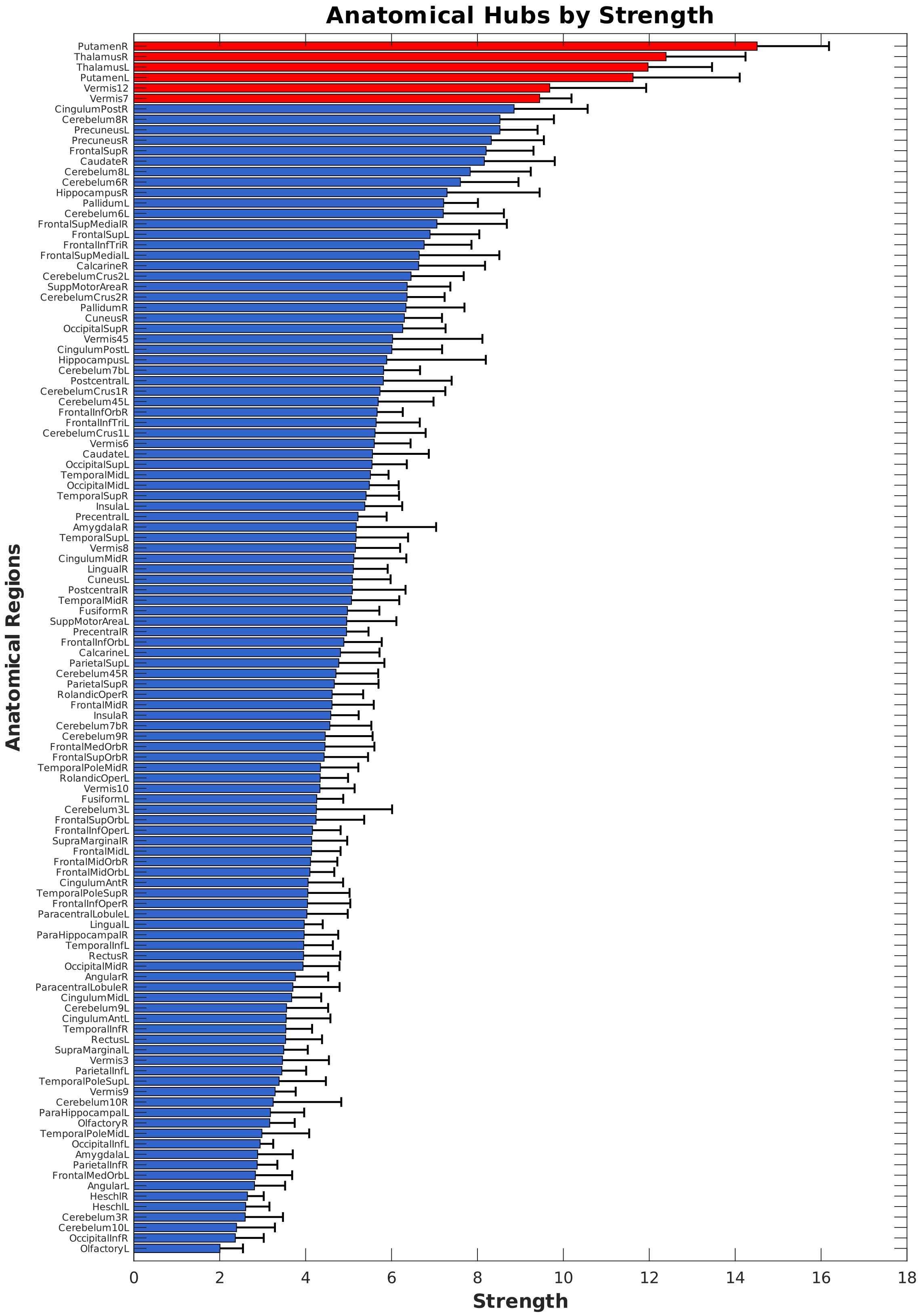

Supplementary Figure 7. Average strength and stnadard deviation of all 116 nodes in proportional threshold analysis. Nodes are ordered by decreasing average strength across all Control datapoints (n=12). Definitions of anatomical regions are in Supplementary Table 1. The top 5% of hubs are visualised in red.

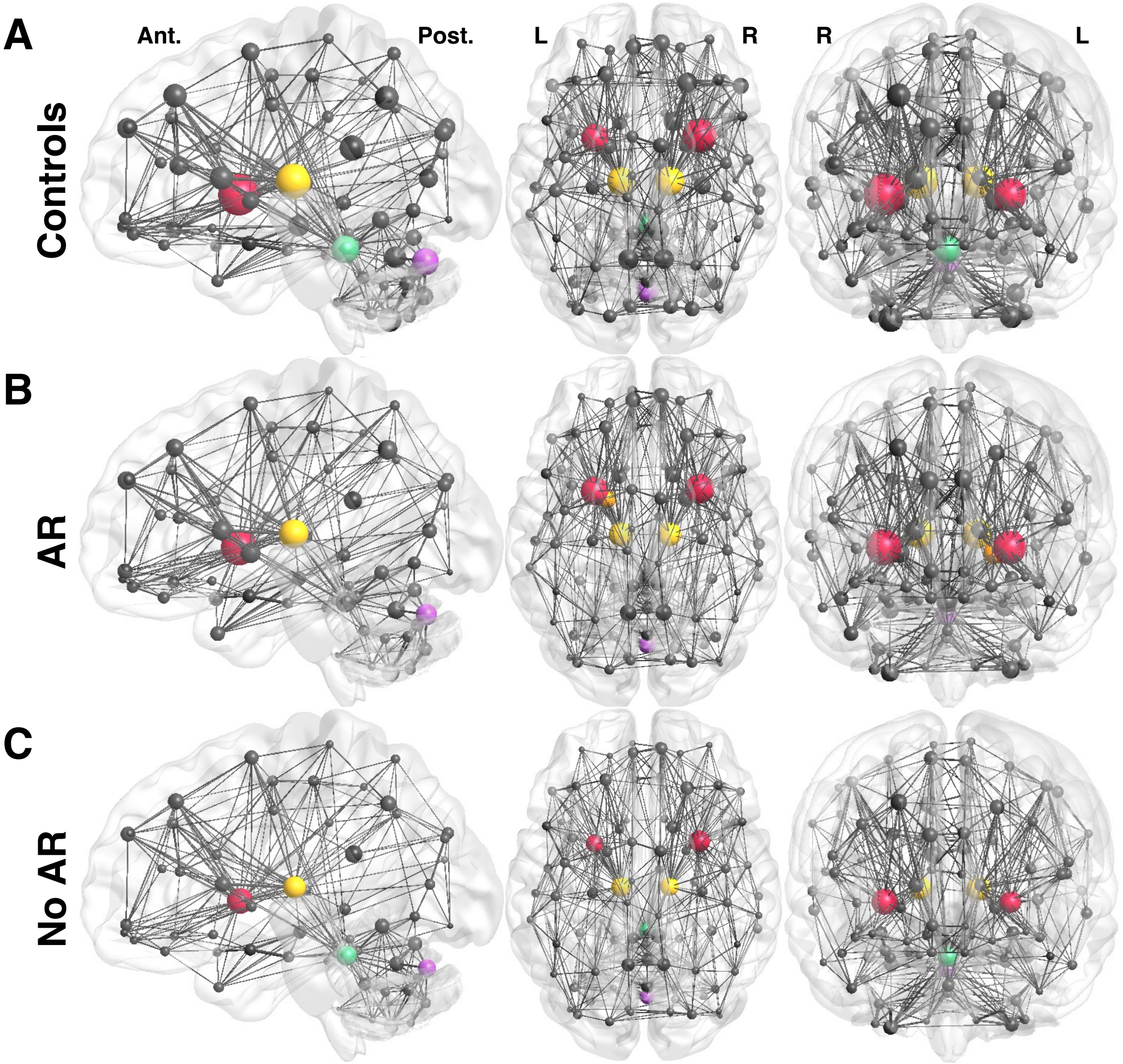

Supplementary Figure 8. Visualisation of the average structural connectome for (A) Controls (n=12), (B) AR patients (n=10) and (C) No AR patients (n=8) using proportional thresholding. The top 5% of hubs (using an proportional threshold of 0.08) are shown (red: putamen, yellow: thalamus, green: vermis lobules I/II, purple: vermis lobule VII, orange: left pallidum [seen in AR]). The size of the nodes scale with their strength. Ant: anterior; AR: arousal recovery, L: left; No AR: No arousal recovery; Post: posterior; R: right.

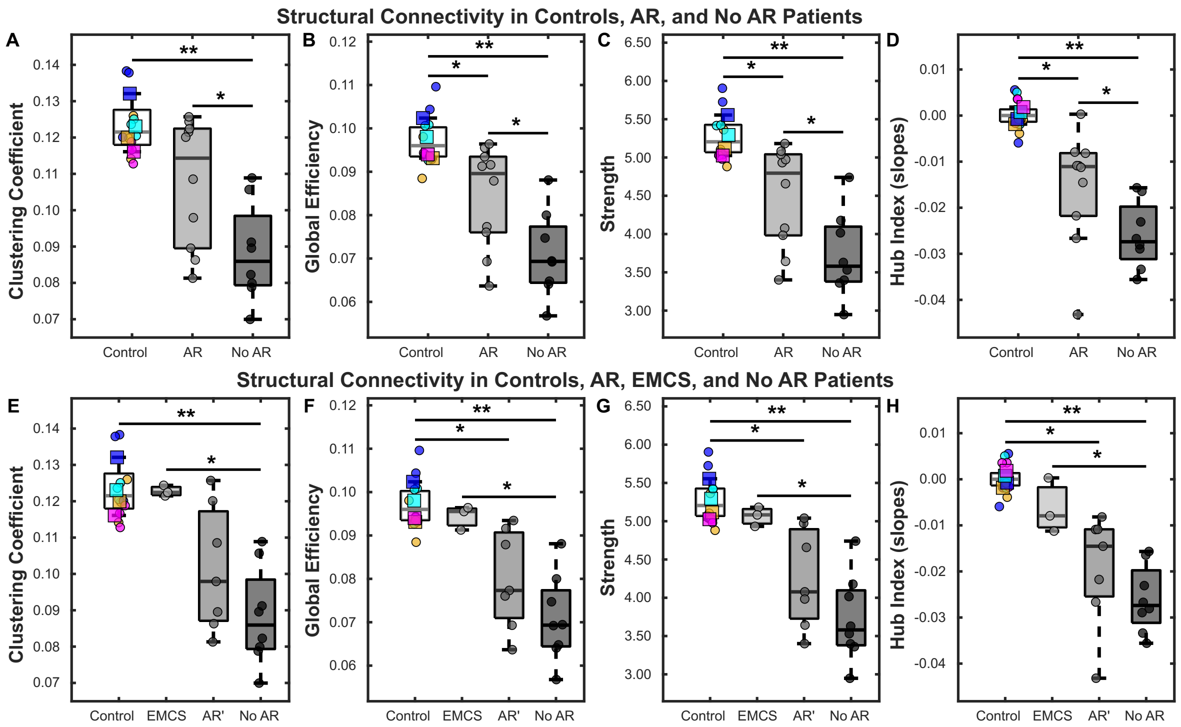

Supplementary Figure 9. Group-level differences in graph theory measures for proportional threshold analysis. Differences in the graph theory measures for (A) clustering coefficient, (B) global efficiency, (C) strength and (D) hub index across Controls (n=4), AR patients (n=10), and No AR patients (n=8). Results separating AR patients who achieved EMCS (n=3) from patients who did not (AR') (n=7) for (E) clustering coefficient, (F) global efficiency, (G) strength and (H) hub index. Statistical comparisons were performed using the Kruskal–Wallis rank-sum test (χ^2 reported) followed by post-hoc two-sample Wilcoxon rank-sum tests (approximate method). χ^2 and p-values for each comparison are provided in Supplementary Tables 3 and 4.*p<0.05. **p<0.01. Individual participant data are shown as filled circles. Mean repeated measures for control subjects are shown as squares. Control data are colour coded by participant (magenta, orange, cyan, blue). AR: Arousal recovery; AR': Arousal recovery patients who did not emerge from a minimally conscious state; EMCS: Emergence from a minimally conscious state; No AR: No arousal recovery.

Supplementary References

1. Dhakal K, Rosenthal ES, Kulpanowski AM, et al. Increased task-relevant fMRI responsiveness in comatose cardiac arrest patients is associated with improved neurologic outcomes. *J Cereb Blood Flow Metab*. Jan 2024;44(1):50-65. doi:10.1177/0271678X231197392
2. Tzourio-Mazoyer N, Landeau B, Papathanassiou D, et al. Automated Anatomical Labeling of Activations in SPM Using a Macroscopic Anatomical Parcellation of the MNI MRI Single-Subject Brain. *Neuroimage*. 2002;15(1):273-289. doi:10.1006/nimg.2001.0978
3. Rubinov M, Sporns O. Complex network measures of brain connectivity: Uses and interpretations. *Neuroimage*. 2010;52(3):1059-1069. doi:10.1016/j.neuroimage.2009.10.003
